# Genotoxic antibody-drug conjugates combined with BCL-XL inhibitors enhance therapeutic efficacy in metastatic castration-resistant prostate cancer

**DOI:** 10.1172/JCI200438

**Published:** 2026-06-23

**Authors:** Galina Semenova, Sander B. Frank, Ruth Dumpit, Wanting Han, Ilsa Coleman, Roman Gulati, Canan D. Dirican, Tarana Arman, Jessica Maruwan, Colm Morrissey, Michael C. Haffner, Peter S. Nelson, John K. Lee

**Affiliations:** 1 Division of Hematology/Oncology, Department of Medicine, Jonsson Comprehensive Cancer Center, UCLA, Los Angeles, California, USA.; 2 Human Biology Division and; 3 Division of Public Health Sciences, Fred Hutchinson Cancer Center, Seattle, Washington, USA.; 4 Department of Urology, University of Washington School of Medicine, Seattle, Washington, USA.; 5 Department of Laboratory Medicine and Pathology, University of Washington, Seattle, Washington, USA.; 6 Division of Clinical Research, Fred Hutchinson Cancer Center, Seattle, Washington, USA.; 7 Department of Medicine, University of Washington, Seattle, Washington, USA.; 8 Parker Center for Cancer Immunotherapy at UCLA, Los Angeles, California, USA.

**Keywords:** Immunology, Oncology, Cancer immunotherapy, Drug screens, Prostate cancer

## Abstract

Metastatic castration-resistant prostate cancer (mCRPC) is an aggressive subtype of prostate cancer (PC) without curative treatments. Antibody-drug conjugates (ADCs) emerged as promising cancer therapeutics that selectively deliver cytotoxic agents (payloads) to the tumors. Although ADCs have been successfully applied to treat hematological and solid tumors, ADC monotherapy has not demonstrated durable responses in mCRPC, and mechanisms of PC resistance to ADCs have not been thoroughly investigated. Our study aimed to improve ADC efficacy using an integrated approach for a custom ADC design and multiplexing. To nominate rational combinations of ADC targets and payloads, we (a) examined protein coexpression of 3 clinically relevant surface antigens — B7-H3, PSMA, and STEAP1 — in human mCRPCs and (b) screened established ADC payloads and their combinations in mCRPC cell lines with different molecular backgrounds. Identified synergistic interactions between DNA-damaging payloads and the BCL-XL inhibitor A-1331852 as well as their coordinated induction of the intrinsic apoptosis pathway were evaluated in PC cell lines. Functional relevance between isolated p53 loss and PC responses to 3 genotoxic ADCs — B7-H3–seco-DUBA, PSMA-SG3249, and STEAP1-DXd — and their combinations with A-1331852 were established using genetic knockout models. Lastly, enhanced in vivo antitumor activity of vobramitamab duocarmazine by systemic A-1331852 was shown. Collectively, our findings provide rationale for development of ADC therapies combining genotoxic payloads with BCL-XL inhibitors for mCRPC.

## Abstract 2

The study presents a framework for combinations of antibody-drug conjugate targets and payloads that may be applied to improve the treatment of late-stage prostate cancer.

## Graphical abstract

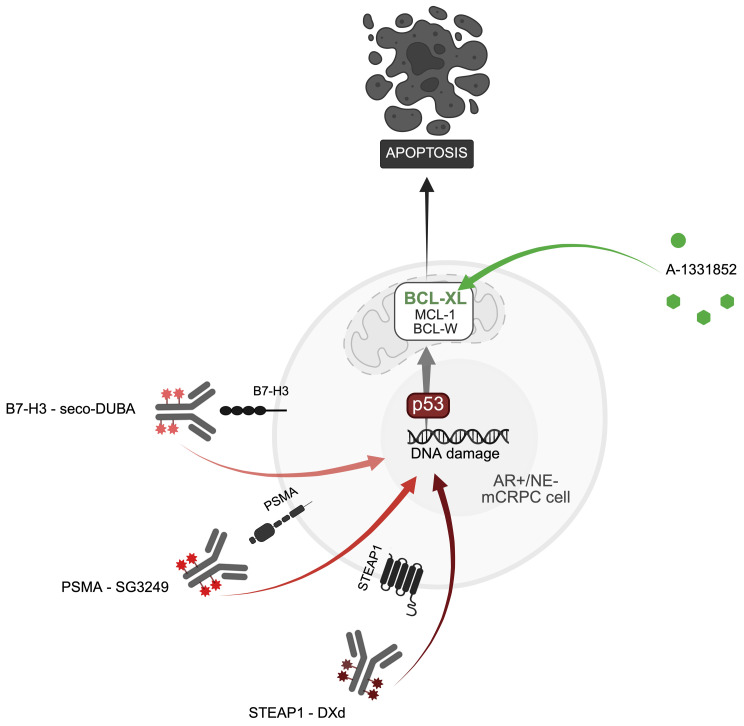

## Introduction

Prostate cancer (PC) is the most frequently diagnosed noncutaneous malignancy and the second leading cause of cancer death in males (1). Localized PC is readily treatable by surgery or radiation therapy; however, metastatic PC remains incurable. While these tumors initially respond to androgen deprivation therapy, nearly all will progress to lethal, metastatic castration-resistant PC (mCRPC). Effective application of systemic chemotherapy to eradicate mCRPC is restricted by dose-limiting toxicities and the inevitable development of resistance (2, 3), suggesting the need for improved drug delivery to the tumor site and diversification of applied cytotoxic agents.

Antibody-drug conjugates (ADCs) are a new generation of targeted therapeutics that combine the high specificity of monoclonal antibodies with the potency of chemotherapeutic drugs (4). An ADC comprises 3 parts: (a) an antibody that binds to an antigen on the target cell surface, (b) a cytotoxic small molecule (payload), and (c) a linker that enables conjugation of the payload to the antibody and plays a critical role in the release of the payload upon delivery to the tumor site (5). These weaponized antibodies can be tailored for specific cancer types, and many ADCs have demonstrated activity against treatment-refractory cancers (4).

Despite the success of ADCs in the treatment of breast and urothelial tumors, the clinical application of ADCs to mCRPC has thus far been unsuccessful (6). Limited responses to ADC monotherapy in mCRPC may be explained by tumor antigen expression heterogeneity, by on-target and off-target systemic toxicity, and by development of tumor resistance to ADC components (6, 7). Under continuous treatment pressure, cancer cells can acquire resistance to both antibody components by downregulation/mutation of the targeted cell surface antigen and to the ADC payload, for example, through the upregulation of drug-efflux transporters (7). Therefore, the rational selection of ADC targets and linker-payloads as well as the development of synergistic ADC combinations have the potential to enhance the therapeutic window for the effective treatment of mCRPC.

To date, several preclinical and clinical studies have assessed the efficacy of ADCs combined with other types of anticancer drugs, including endocrine therapy, chemotherapy, targeted molecular agents, and immunotherapy for the treatment of solid tumors (8). These combination therapies have demonstrated some success in improving antitumor efficacy; however, research on PC combination therapies involving ADCs remains limited.

In this study, we evaluated the benefits of ADC combinations that leverage synergism between 2 distinct and complementary ADC payloads. To design ADCs for combination mCRPC therapy with improved efficacy and specificity, we first analyzed the distribution of clinically relevant PC targets — prostate-specific membrane antigen (PSMA; *FOLH1*) (9), 6-transmembrane epithelial antigen of prostate-1 (STEAP1) (10, 11), and B7 homolog 3 (B7-H3; *CD276*) (12–14) — in mCRPC and normal tissues and proposed to use these antigens in pairs for dual ADC targeting. Next, we screened established ADC payloads in pairwise combinations and identified synergistic interactions between DNA-damaging payloads and the BCL-XL inhibitor A-1331852. Finally, we confirmed the activity of synergistic payloads administered through combination ADC therapy using in vitro and in vivo PC models. Collectively, our findings indicate that combining ADCs that enable simultaneous delivery of 2 synergistic payloads is a promising approach to potentiate therapeutic activity in mCRPC.

## Results

### B7-H3(CD276), PSMA(FOLH1), and STEAP1 are coexpressed in metastatic PCs.

We sought to enhance the antitumor effects of PC therapeutics by directing the cytotoxic payloads via antibody delivery and using combination strategies that exploit multiple target antigens and synergistic payloads. Prior findings show that coadministration of 2 ADCs targeting the same antigen may lead to binding competition and reduced efficiency of payload delivery (15). Thus, we proposed to use 2 different antigens to simultaneously cotarget tumor cells with ADCs for the treatment of mCRPC. Such target pairs should be expressed on the surface of the same PC cells though have limited coexpression in normal tissues.

**Figure 1:**
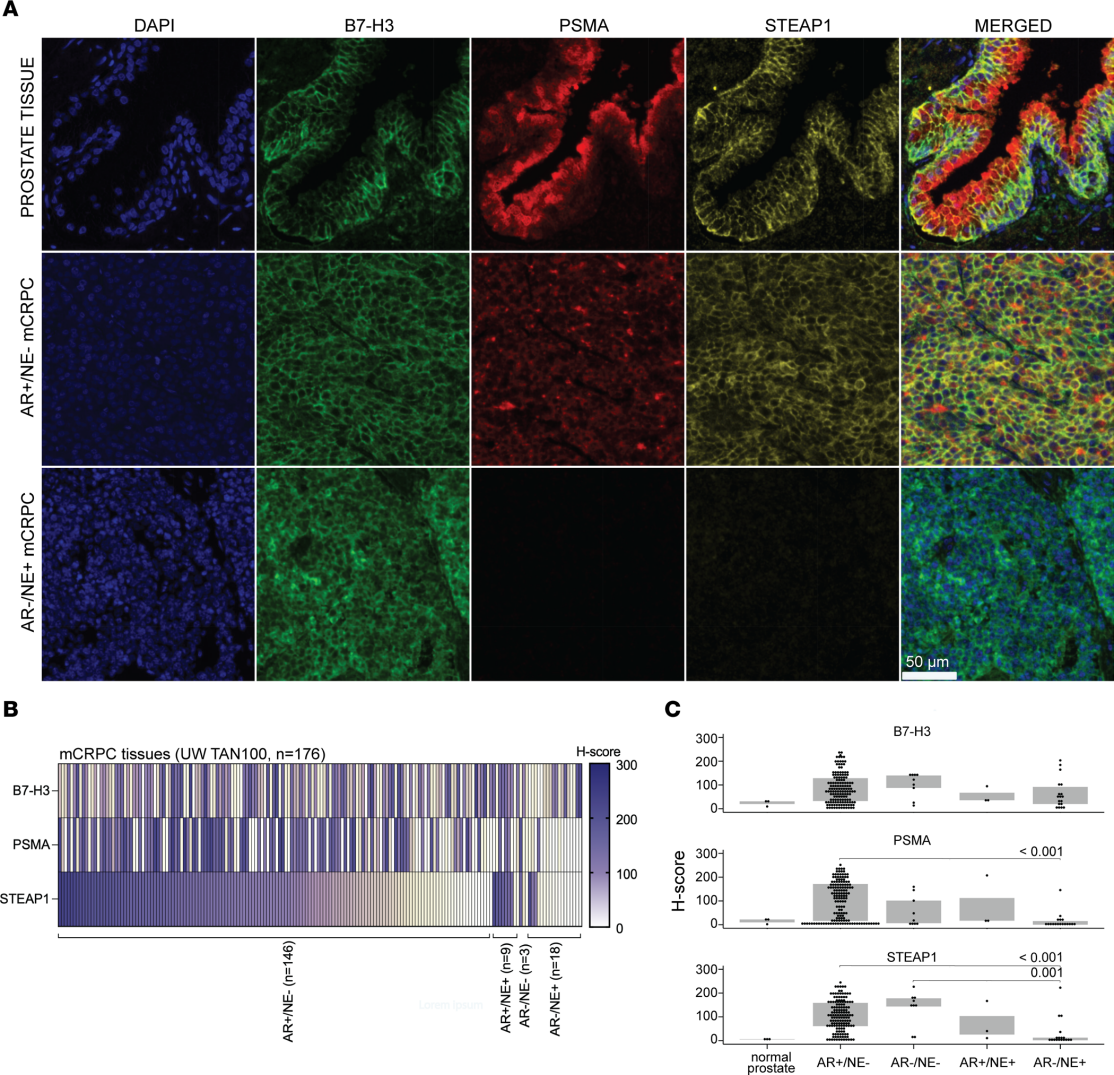
B7-H3, PSMA, and STEAP1 are expressed in the majority of AR-regulated mCRPCs. (**A**) Representative TMA images of human prostate tissue (FDA999 L206) and mCRPC tissues (UW TAN100) with membranous B7-H3, PSMA, STEAP1, and nuclear DAPI staining. Scale bar: 50 �m. (**B**) Heatmap of individual H-scores of 176 mCRPC tissues. (**C**) Averaged H-scores of normal prostate, AR^+^/NE^–^, AR^+^/NE^+^, AR^–^/NE^–^, and NEPC tissue samples. Wilcoxon-Mann-Whitney rank-sum test (2-sided) *P* values are shown. *P* values were corrected for multiple comparisons using Holm’s method.

**Figure 2:**
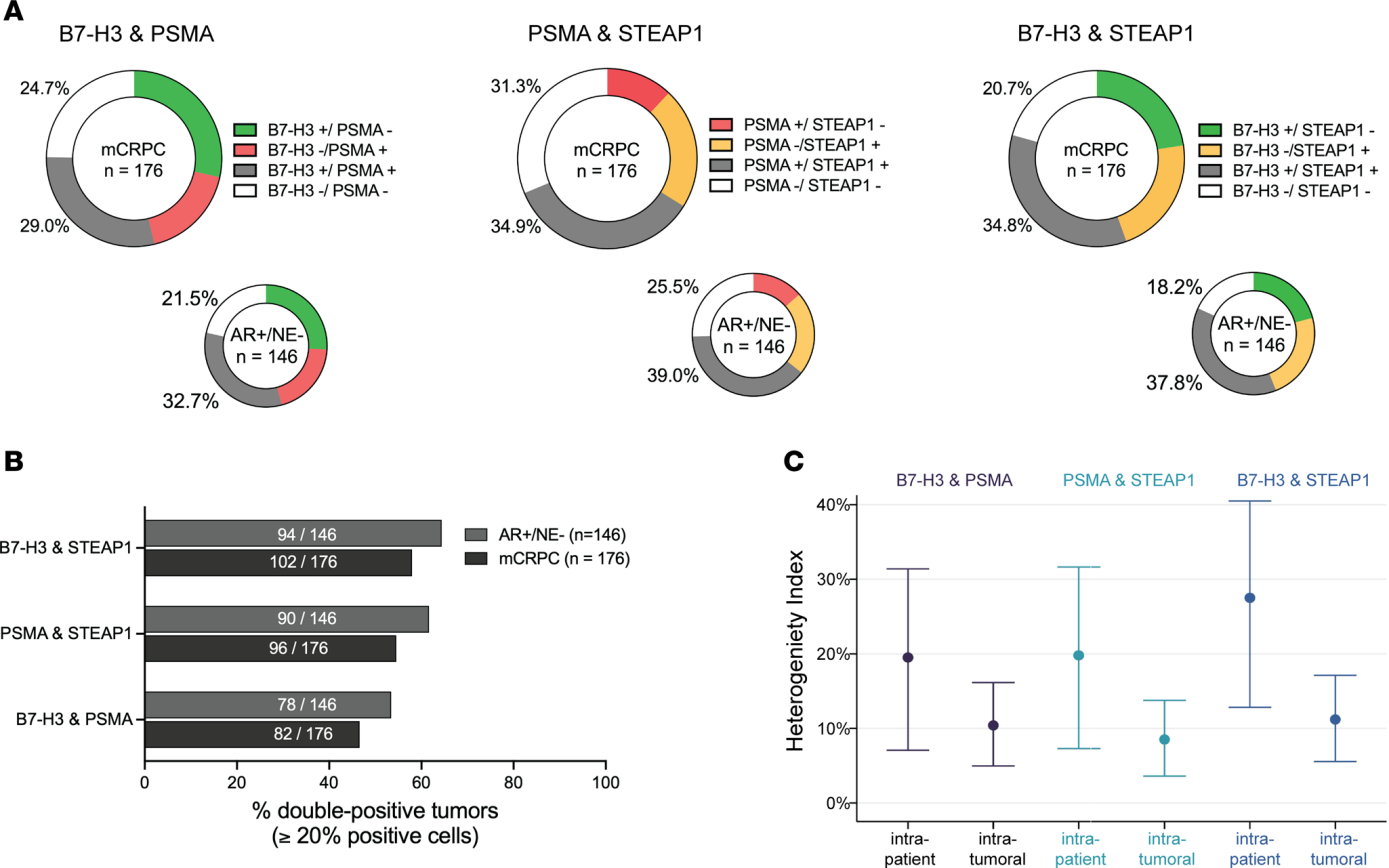
mCRPC cells coexpress ADC target pairs PSMA and STEAP1, B7-H3 and PSMA, and B7-H3 and STEAP1. (**A**) Averaged percentage double-positive and -negative cells in all mCRPC and specifically in AR^+^/NE^–^ tumors. (**B**) The percentage of mCRPC or AR^+^/NE^–^ tumors costaining for each antigen pair. (**C**) Hypergeometric mean (95% CI) of antigen coexpression heterogeneity indices across different metastatic sites in a given patient (intrapatient heterogeneity) and within a metastatic site (intratumoral heterogeneity).

Several surface antigens have been shown to be highly expressed by PCs and were previously employed as ADC targets for mCRPC. The most well-established antigens include PSMA (16, 17), STEAP1 (10, 11, 18), and B7-H3 (14, 19); however, to exploit combinatorial effects, it is essential to confirm that these antigens are expressed by the same cells in mCRPCs. To address this issue, we conducted multiplexed immunofluorescence (mIF) staining of a clinically and histologically annotated case series of mCRPC tumors for B7-H3, PSMA, and STEAP1 (Figure 1A and Supplemental Table 3; supplemental material available online with this article; https://doi.org/10.1172/JCI200438DS1). Tumor tissues were collected at rapid autopsy through the University of Washington Tissue Acquisition Necropsy (UW TAN) program (20). Tumor sections were organized within a tissue microarray (TMA) comprising 181 tumors from 58 patients. Five tumors were not analyzed due to insufficient tumor content, leaving 176 evaluable tumors (514 individual cores) from 58 patients. Tumors were classified into 4 phenotypic subtypes based on immunohistochemical staining for androgen receptor (AR) signaling and neuroendocrine (NE) markers, as previously described (21, 22): AR-active prostate cancer (AR^+^/NE^–^), amphicrine carcinoma (AR^+^/NE^+^), double-negative mCRPC (AR^–^/NE^–^), and neuroendocrine prostate cancer (NEPC; AR^–^/NE^+^). Integrated H-scores combining the frequency and intensity of staining were used to evaluate single-antigen staining (Figure 1). For dual-antigen staining analysis, quantification of the percentage of double-positive cells was applied (Figure 2).

We observed B7-H3, PSMA, and STEAP1 staining heterogeneity across the 4 mCRPC phenotypic groups (Figure 1B). Consistent with prior reports (11, 16), STEAP1 and PSMA H-scores were significantly greater in AR^+^/NE^–^ tumors compared with NEPC; however, B7-H3 reactivity was observed in both AR-active and NE tumors (Figure 1C). These findings are consistent with the corresponding B7-H3/*CD276*, PSMA/*FOLH1*, and *STEAP1* gene expression analysis in 3 cohorts determined by RNA-seq: 270 Stand Up To Cancer (SU2C) international dream team mCRPC biopsies, 172 mCRPC samples from the UW TAN program, and 126 LuCaP PC patient-derived xenograft (PDX) samples (Supplemental Figure 1).

B7-H3, PSMA, and STEAP1 IF staining was evaluated pairwise at single-cell resolution, within tumors, and across tumors for individual patients. To assess the fraction of the cells coexpressing 2 antigens, the percentage of double-positive cells was calculated in all tumor specimens (Figure 2A). We determined that approximately one-third of all mCRPC cells stained positively for the combination of PSMA and STEAP1, B7-H3 and PSMA, or B7-H3 and STEAP1. Within the AR^+^/NE^–^ mCRPC cohort 32.7%–39% of cells were double-positive. The largest antigen overlap was observed between PSMA and STEAP1, where 39% of cells comprising AR^+^/NE^–^ tumors stained for both, while the greatest antigen coverage was achieved with the B7-H3 and STEAP1 pair, with only 18.2% cells in AR^+^/NE^–^ tumors staining for neither antigen (81.8% cells stained for either B7-H3 or STEAP1). Overall, 20% of mCRPC cells across all phenotype groups and 25% of AR^+^/NE^–^ cells stained positively for all 3 antigens.

We next determined how many tumors contained a large fraction of double-positive cells. Tumor cores with ≥20% cells costaining for any antigen pair were considered double-positive. About half of the tumors were double-positive for at least 1 antigen pair, and most of the tumors were B7-H3 and STEAP1 positive: 58% (102/176) mCRPCs and 64.4% (94/146) AR^+^/NE^–^ tumors (Figure 2B and Supplemental Figure 2, A and B). The B7-H3 and STEAP1 pair also demonstrated the highest estimated staining heterogeneity (27.4% intrapatient and 11.2% intratumoral), followed by B7-H3 and PSMA (19.5% and 9.4%), and PSMA and STEAP1 (19.4% and 8.4%) (Figure 2C). Of the mCRPC patients, 46/58 had at least 1 B7-H3 and STEAP1 positive tumor, 39/58 patients demonstrated at least 1 PSMA and STEAP1 positive tumor, and 34/58 patients harbored at least 1 B7-H3 and STEAP1 positive tumor (Supplemental Figure 2C). All tumors stained positive for STEAP1 and B7-H3 in 17 patients, for PSMA and STEAP1 in 21 patients, and for B7-H3 and PSMA in 15 patients. Collectively, these assessments of intratumor and intraindividual heterogeneity provide data to inform combinatorial strategies designed to target the same or divergent cell populations within a patient and how to deploy targeted agents with systemic delivery.

The 20% coexpression cutoff was initially selected as a moderate threshold to account for tumor heterogeneity. Importantly, tumors with ≥20% double positivity exhibited up to 100% total positivity for the individual antigens (Supplemental Figure 2B and Supplemental Table 3). Antigen thresholds used in the clinic for antibody-based therapies are highly variable and drug specific; for example, ADCs with membrane-permeable payloads can achieve efficacy at low antigen levels (23), whereas others require higher antigen density to ensure efficient internalization and payload delivery (24). To provide a more comprehensive characterization, we also conducted antigen coexpression analyses at 1% and 50% thresholds to capture the spectrum of clinically relevant expression levels, reflecting both maximum bystander potential and stringent requirements for high-density targeting (Supplemental Figure 2D).

Antigen target coexpression was also evaluated in a human benign tissue TMA (Figure 1A and Supplemental Figure 3). We found that luminal cells of the prostate and mammary gland stained positively for all 3 antigens: STEAP1, B7-H3, and PSMA. Kidney tubular cells and small intestine enterocytes costained for STEAP1 and PSMA, while urothelial cells and colon enterocytes showed low intensity B7-H3 and STEAP1 staining.

Overall, these data collectively show that each of the 3 antigen pairs demonstrates high levels of double positivity in mCRPC tumors, particularly in the AR^+^/NE^–^ phenotype, which is the most common subtype of mCRPC, and very low levels of double positivity in normal tissues, suggesting that these antigen pairs may be compelling targets for combination ADC therapy.

### Nomination of synergistic interactions between established ADC payloads in PC.

To date, the majority of ADCs comprise payloads with similar mechanisms of action (MoAs) that fall into 2 major classes: microtubule targeting and DNA-damaging agents. This raises concerns about the development of cross-resistance with systemic standard-of-care treatments or with other ADCs if used sequentially. We therefore asked if a rational selection of the payload MoA for a specific molecular subtype of PC could improve therapeutic response and mitigate ADC resistance.

Recent efforts have yielded ADC payloads using different MoAs beyond conventional agents targeting DNA or microtubules (25). The development of novel payloads provides opportunities for ADC cotargeting, as combinations of agents with different MoAs are predicted to have the highest probability of overcoming resistance without overlapping toxicities. Payload combinations that offer additive or synergistic cytotoxic effects on cancer cells can potentially increase ADC efficacy and reduce effective therapeutic doses.

**Figure 3:**
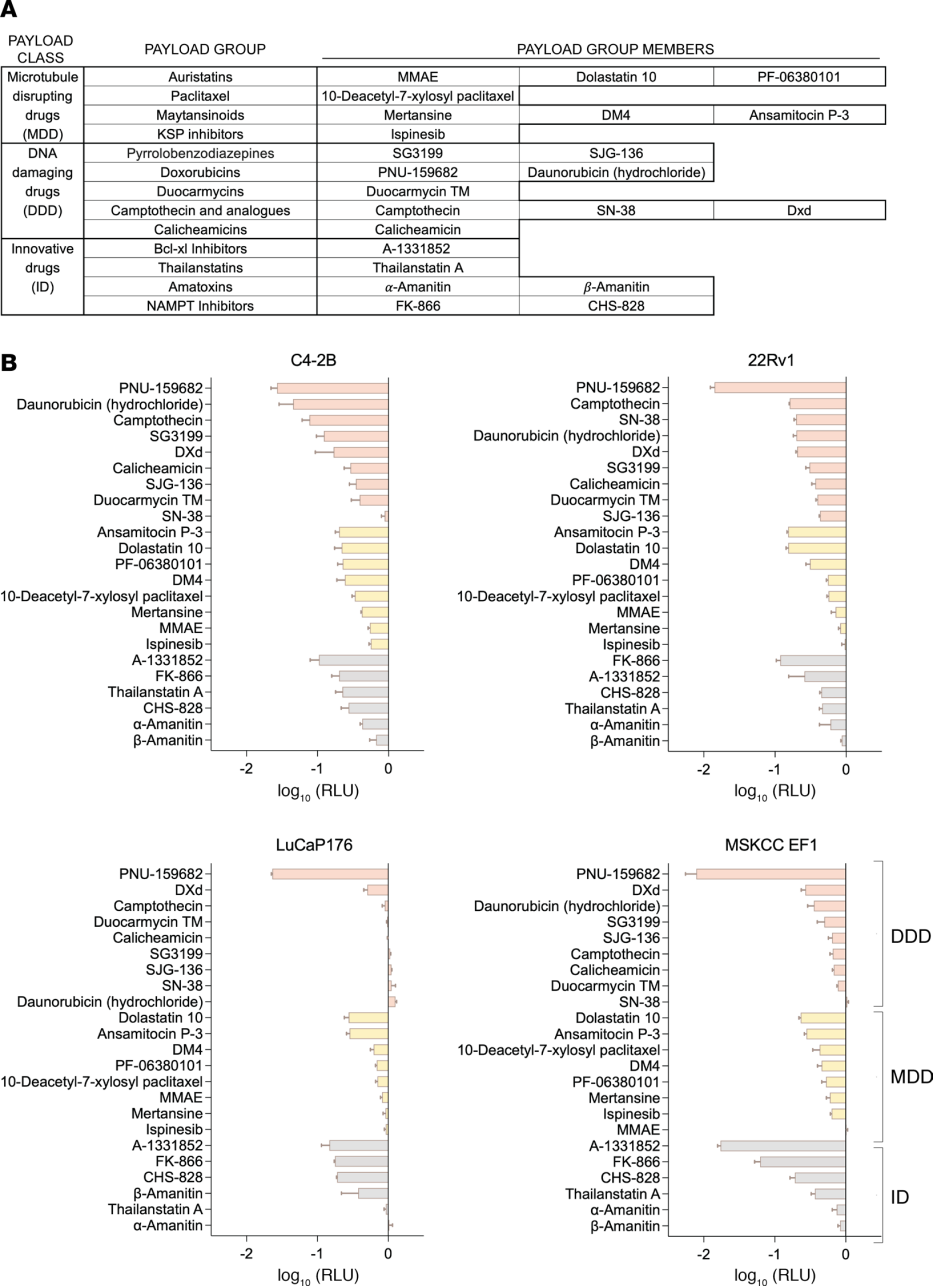
Screening of ADC payloads in PC cell lines. (**A**) List of cytotoxic agents tried in the drug screen. (**B**) Relative viability of PC cells exposed to the screen compounds (low dose): 10-deacetyl-7-xylosyl paclitaxel (0.5 μM), PF-06380101 (0.1 nM), mertansine (0.1 nM), DM4 (0.5 nM), ispinesib (0.5 nM), SG3199 (0.1 nM), SJG-136 (0.5 nM), PNU- 159682 (0.1 nM), daunorubicin (hydrochloride) (0.1 μM), DXd (0.1 μM), calicheamicin (0.1 nM), thailanstatin A (0.1 μM), duocarmycin TM (0.1 nM), ansamitocin P-3 (0.5 μM), camptothecin (0.1 μM), dolastatin 10 (0.1 μM), A-1331852 (0.5 μM), α-amanitin (50 nM), β-amanitin (50 nM), FK-866 (50 nM), CHS-828 (5 nM), SN-38 (10 nM), and MMAE (0.1 nM). (**B**) Normalized responses of C4-2B, 22Rv1, LuCaP176, and MSKCC EF1 to individual payload exposure (low dose). Compounds are organized by the payload class: DDDs (salmon), MDDs (yellow), and IDs (gray). Results are expressed as log_10_ RLUs, measured via the CellTiter-Glo luminescent assay as a marker of total viability.

To evaluate the PC cell response to individual ADC payloads and to nominate synergistic payload interactors, we designed a drug screen with 23 small molecules representing 3 major classes of ADC payloads: microtubule-disrupting drugs (MDDs), DNA-damaging drugs (DDDs), and innovative drugs (IDs) targeting RNA synthesis (thailanstatins, amatoxins), NAD synthesis (NAMPT inhibitors), or apoptosis evasion (BCL-XL inhibitors). Each payload class included 4–5 pharmacological groups with different MoAs and 1–3 analogous members per group (Figure 3A). The 23 agents were evaluated at 2 working concentrations (high and low), which were individually selected for each compound based on effective concentrations reported prior (Figure 3A and Supplemental Table 1). The high concentration was chosen based on published working concentrations and IC_50_ ranges in other cancer models for these agents (26–44), and the low concentration represented a 10-fold dilution of the high dose. This range was selected to capture activity across sensitive and resistant cell lines. We also evaluated 13 out of 23 compounds (1 representative from each payload group) in pairs at low working concentrations, resulting in 78 total pairwise drug combinations (Supplemental Table 4). Twenty-three payloads and active payload derivatives were tested in 4 mCRPC cell lines representing different phenotypic subtypes: C4-2B (AR^+^/NE^–^), 22Rv1 (AR^+^/NE^+^), LuCaP 176 (AR^–^/NE^–^), and MSKCC EF1 (AR^–^/NE^+^) (Figure 3, A and B, Supplemental Figure 4, and Supplemental Table 4). Agents from all 3 payload classes effectively killed AR-regulated cells C4-2B and 22Rv1 at high and low doses (Figure 3B and Supplemental Figure 4). The LuCaP 176 cell line demonstrated weaker responses to many drugs and in particular to DDDs compared with other lines. The NEPC cell line MSKCC EF1 showed moderate responses to MDDs and DDDs; however, it was sensitive to several IDs, including the BCL-XL inhibitor A-1331852, suggesting potential utility of this payload in the development of ADCs for NEPC. When administered at high doses, cytotoxic effects of DDDs were significantly greater than the effects of MDDs across all mCRPC models (Supplemental Figure 4).

**Figure 4:**
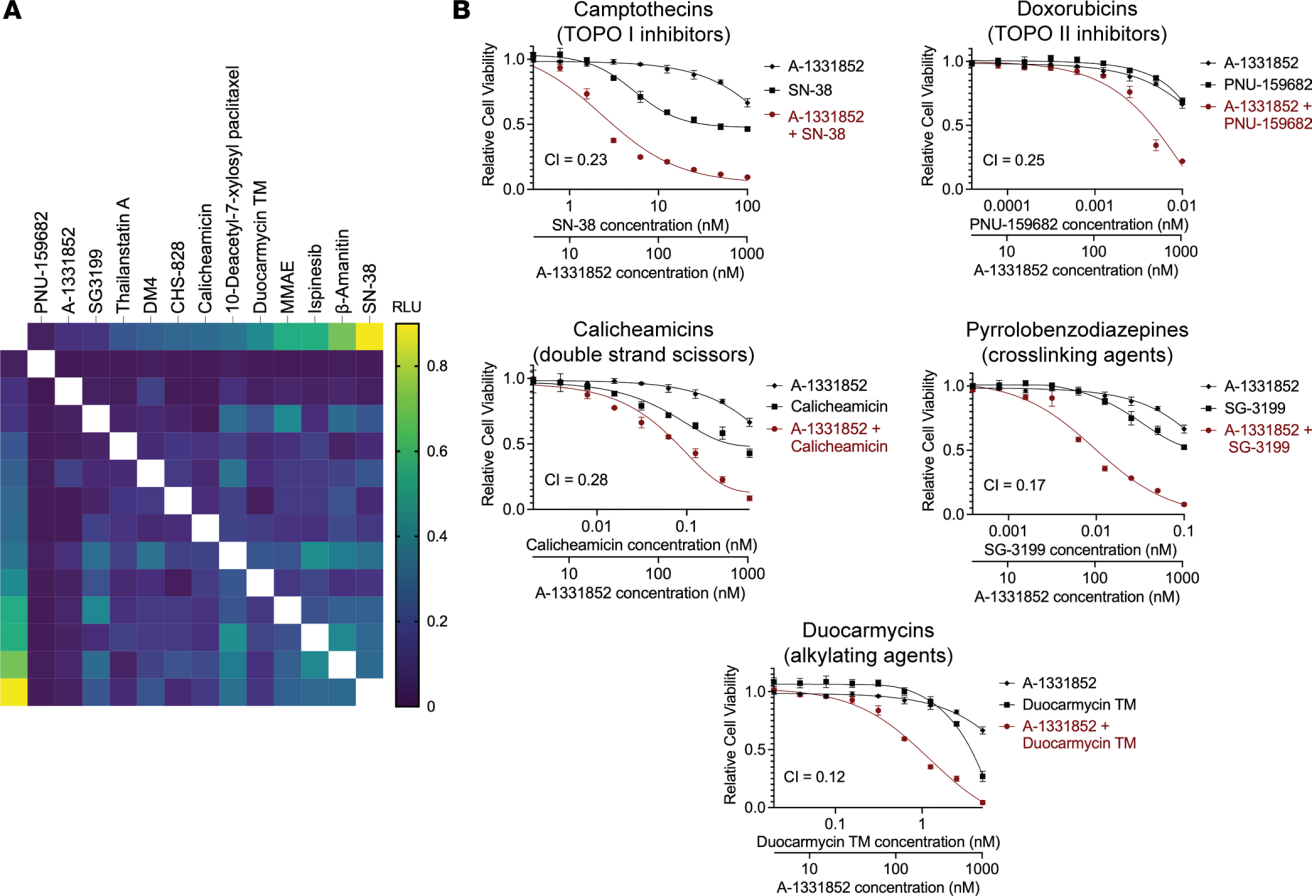
Combinatorial drug screening identifies synergistic interactors. (**A**) Heatmap demonstrating normalized responses of C4-2B cells to single payloads (data points on the left and upper edge) and payload pairs (data points in the center of the heatmap). (**B**) Dose response to BCL-XL inhibitor A-1331852; DDDs SN-38, PNU-159682, calicheamicin, SG-3199, and Duocarmycin TM; and 5 combinations of DDDs with A-1331852 in LNCaP cells. CI, combination index.

We next assessed the effects of drug/payload combinations. We observed marked differences in the response to drug combinations among the 4 PC models (Supplemental Figure 5A). Payload pairs for the synergy validation were selected from the interactors with different MoAs that exhibited PC cell toxicity greater than the amplification of their individual effects in at least 2 cell lines (Figure 4A and Supplemental Figure 5A). Potential synergistic interactions were observed between DDDs and IDs, as well as between the members of the ID group (Supplemental Figure 5B). These pairs were prioritized and further validated using a dilution series of payloads alone and in combination. Drug additivity or synergism between payloads was analyzed in the in vitro cell viability assays using the Chou-Talalay method (45). Interactors with combination indices < 1.0 were considered synergistic. Based on these validation studies, a highly enriched group of synergistic interactions was identified between the BCL-XL inhibitor A-1331852 and DNA-damaging agents from all 5 DDD groups tested in the screen: SN-38 (TOPO I inhibitor), PNU-159682 (TOPO II inhibitor), calicheamicin (double-stranded DNA scissor), SG-3199 (DNA cross-linking agent), and duocarmycin TM (DNA alkylating agent) (Figure 4B).

To confirm on-target drug effects, synergism between duocarmycin TM and A-1331852 was further verified using the drug analogs WEHI-539 (BCL-XL inhibitor) and seco-DUBA (duocarmycin analog) in a panel of 8 PC cell lines (Supplemental Figures 6 and 7, and Supplemental Table 5). All but 1 of the 8 models tested demonstrated a combination index of < 1.0 with each combination. The 22Rv1 model was the exception, and these results can likely be attributed to the low BCL-XL protein level in this cell line (Supplemental Figure 8), as BCL-XL (*BCL2L1*) expression may be necessary for the cell response to the proposed payload combination. These findings suggest that cotargeting mCRPC with 2 ADCs bearing DNA-damaging agent and BCL-XL inhibitor as payloads may improve tumor responses to ADC therapy.

### Induction of p53-mediated apoptosis through DNA damage and BCL-XL inhibition in PC.

The synergism between DDDs and A-1331852 can be explained by the interplay between DNA damage and BCL-XL function in the intrinsic p53-dependent apoptosis pathway (46). In response to DNA damage, p53 is rapidly activated and stabilized through posttranslational modifications that engage several cell death programs, including mitochondrial cell death (47). Prosurvival BCL-2 family proteins, including BCL-2, BCL-XL, MCL-1, and BCL-W, are localized in the mitochondria and prevent apoptosis by inhibiting permeabilization of the outer mitochondrial membrane (48). Our previous report (49) and current data indicate that while BCL-2 is overexpressed in mCRPCs with NE phenotype, BCL-XL is abundant in both AR-active tumors and NEPCs (Supplemental Figures 8 and 9). Our analysis of 2 integrated datasets, the Grasso 2012 microarray (Gene Expression Omnibus [GEO]: GSE35988) (50) and the University of Washington (UW) cohort, demonstrates that the expression of BCL-XL/*BCL2L1* is elevated in mCRPC compared with hormone-sensitive prostate cancer, suggesting that castration-resistant tumors rely on this protein for apoptosis evasion (Supplemental Figure 10A). We found that the majority of mCRPCs express high levels of BCL-XL (Supplemental Figure 9), and we did not observe a significant correlation between BCL-XL expression and patient survival or resistance to standard-of-care therapies in the UW mCRPC cohort (Supplemental Figure 10, B and C).

**Figure 5:**
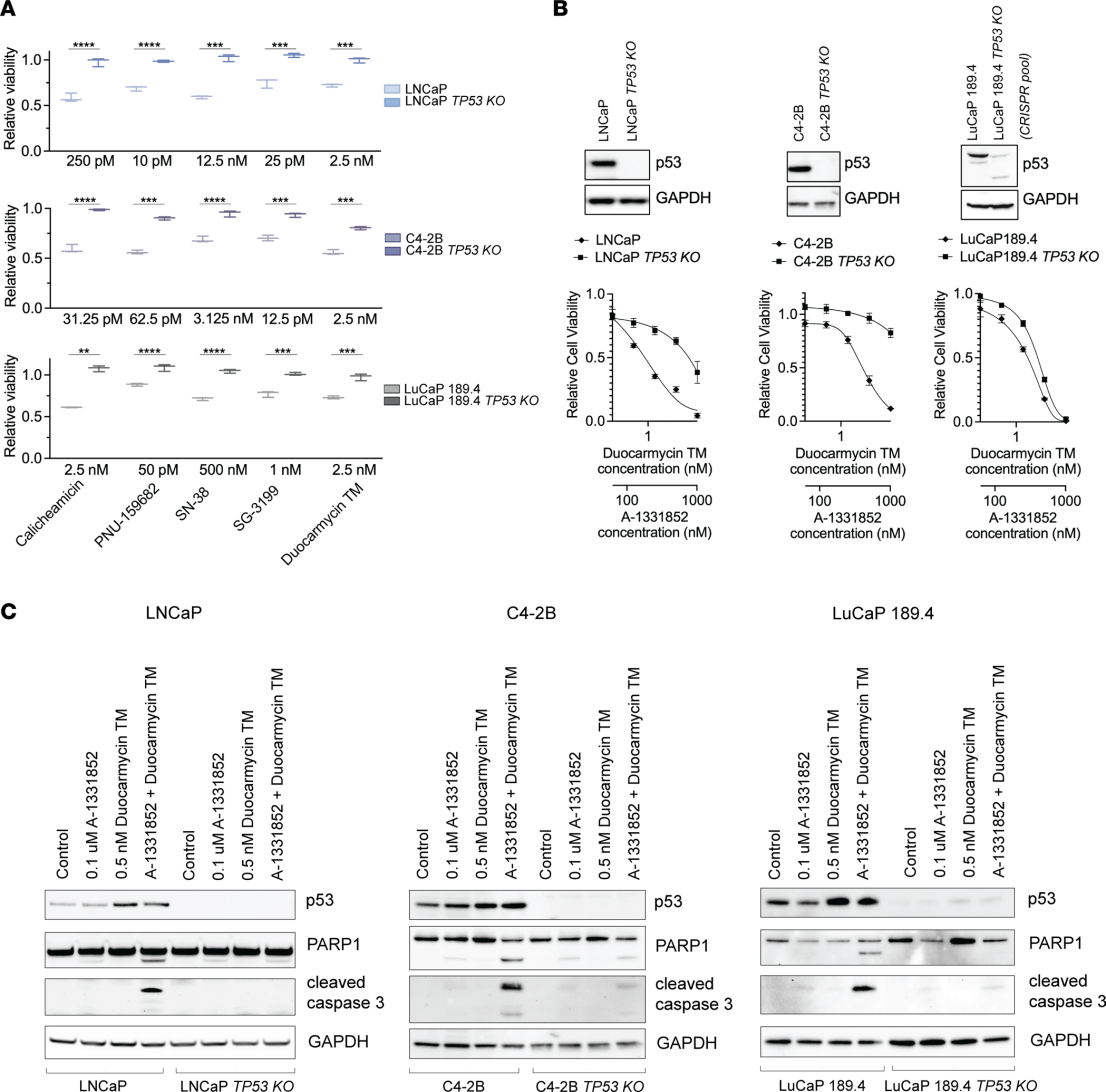
Combination of Duocarmycin TM with A-1331852 induces apoptosis in PC lines expressing p53(*TP53*) and BCL-XL(*BCL2L1*). (**A**) Relative viability of PC cells lines expressing WT *TP53* (LNCaP, C4-2B, LuCaP189.4) and isogenic *TP53-*KO cells exposed to DNA-damaging agents for 72 hours. Data are shown as mean ± SD. Significance was determined using unpaired 2-tailed *t* test. A *P* value < 0.05 was considered significant. **P < 0.01, ***P < 0.001, ****P < 0.0001. (**B**) Immunoblot analysis of p53 protein levels and dose response of isogenic lines to Duocarmycin TM and A-1331852 combination. (**C**) Immunoblot analysis of p53, PARP1, and cleaved caspase-3 in isogenic cell lines treated with DMSO (control), A-1331852, Duocarmycin TM, or the combination of A-1551852 and Duocarmycin TM for 24 hours.

In several cancer types, p53 loss of function promotes resistance to DNA-damaging chemo- and radiotherapeutics (51). In this context, we determined that over a third of AR^+^/NE^–^ tumors in the SU2C cohort demonstrate biallelic loss of *TP53* (Supplemental Figure 9). We queried how functional *TP53* KO would affect PC cell responses to genotoxic payloads and their combinations with the BCL-XL inhibitor A-1331852. To compare the cytotoxicity of DDDs in isogenic cell models, we performed CRISPR/Cas9 KO of *TP53* to generate 3 isogenic PC models with and without functional *TP53*: LNCaP (single clone) (52), C4-2B (single clone), and LuCaP 189.4 (KO pool). Parental and *TP53-*KO cells were treated with SN-38, PNU-159682, calicheamicin, SG-3199, and duocarmycin TM for 72 hours (Figure 5A). In all 3 isogenic cell line pairs, we observed a weaker response to DDDs in *TP53-*KO cells compared with the parental cells with intact *TP53*. Likewise, parental cells were more sensitive to duocarmycin TM and A-1331852 combination (Figure 5B). Induction of apoptosis in p53-proficient cells after a 24-hour exposure to duocarmycin TM and A-1331852 was confirmed by assaying PARP1 and caspase-3 cleavage (Figure 5C). We also observed an increase in p53 protein levels in the p53^+^ cells treated with duocarmycin TM, which is indicative of p53 stabilization following DNA damage (Figure 5C). These findings imply that, along with BCL-XL (*BCL2L1*) expression, p53 proficiency might be a contributing factor to PC cell response to the combination of DDDs and BCL-XL inhibitor payloads.

### Genotoxic ADCs combined with the BCL-XL inhibitor A-1331852 enhance cytotoxicity.

**Figure 6:**
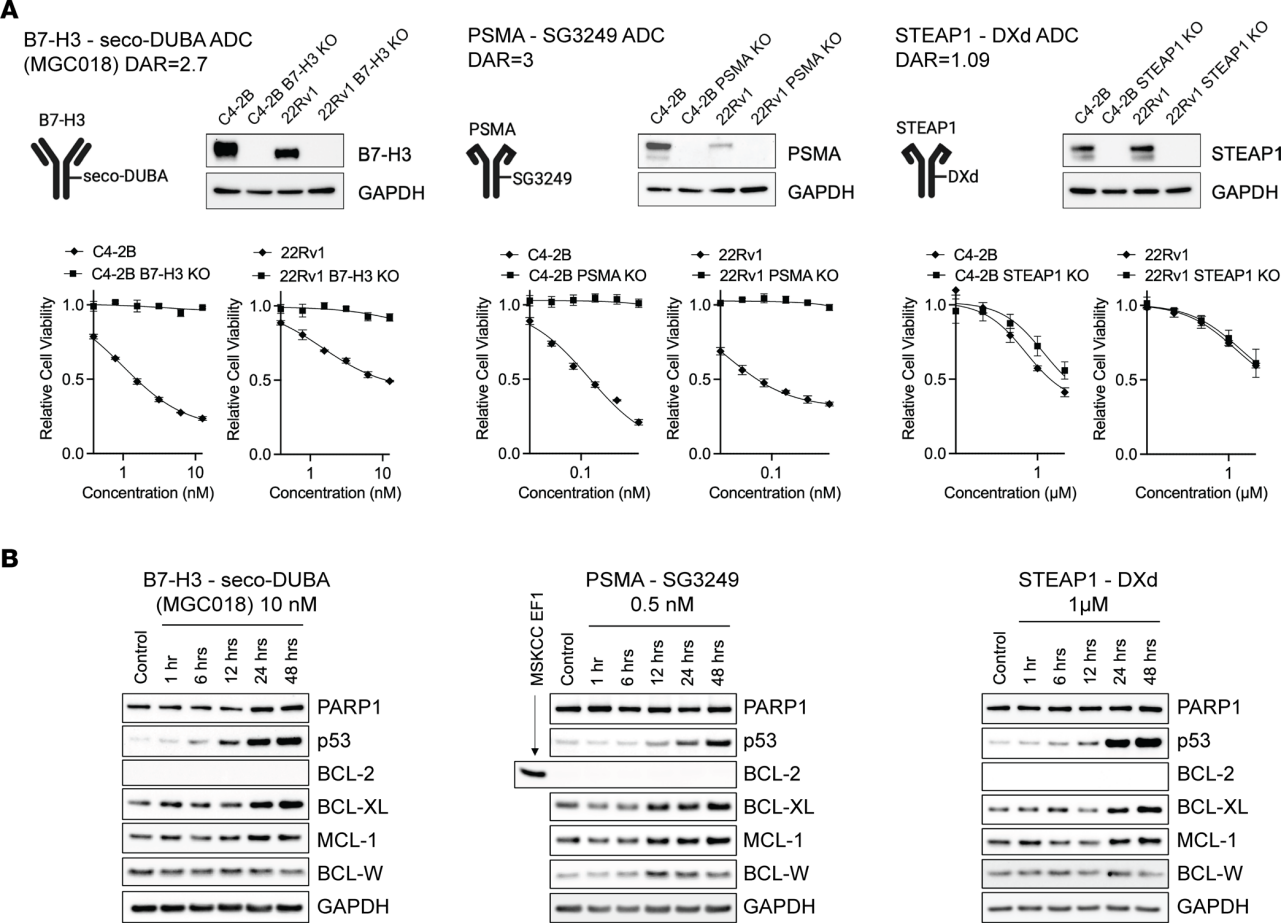
Genotoxic ADCs induce upregulation of anti-apoptotic BCL-2 family proteins. (**A**) Diagrams of B7-H3–seco-DUBA, PSMA-SG3249, and STEAP1-DXd ADCs. Immunoblots showing B7-H3, PSMA, and STEAP1 KOs in isogenic C4-2B and 22Rv1 cell lines. Differential response of antigen-expressing cell lines to B7-H3–seco-DUBA, PSMA-SG3249, and STEAP1-DXd ADCs (4-day exposure). (**B**) Immunoblot analysis of PARP1, P53, BCL-2, BCL-XL, MCL-1, and BCL-W in LNCaP PC cells exposed to B7-H3–seco-DUBA, PSMA-SG3249, or STEAP1-DXd ADCs for 1–24 hours. MSKCC EF1 sample was used to locate the position of the BCL-2 band.

To verify that payload synergism can be translated into ADC combination regimens, we combined ADCs bearing DNA-damaging payloads with unconjugated BCL-XL inhibitor A-1331852. As conjugation of A-1331842 to an antibody required specialized synthetic expertise, this drug was used as a free systemic agent for a proof of concept. Three different genotoxic ADCs targeting B7-H3, PSMA, or STEAP1 were tested singly and in combination with A-1331852 (Supplemental Table 6). C4-2B and 22Rv1 cell lines with intact B7-H3 (*CD267*), PSMA (*FOLH1*), and STEAP1 and isogenic paired lines with gene deletions of these proteins (B7-H3 KO, PSMA KO, and STEAP1 KO) were engineered to confirm antibody specificity and ADC responses (Figure 6A).

B7-H3–seco-DUBA (MGC018, vobramitamab duocarmazine, vobra duo), a clinical-grade ADC targeting B7-H3 and bearing the genotoxic seco-DUBA duocarmycin prodrug payload (19), was provided by MacroGenics. To produce additional genotoxic ADCs, we synthesized single-chain antibodies (scFv-Fc) binding PSMA (J591 biosimilar) and STEAP1 (vandortuzumab biosimilar) (Supplemental Figure 11). The binding specificity of PSMA scFv-Fc was confirmed by flow cytometry in parental and PSMA-KO cells, while specificity of STEAP1 scFv-Fc was confirmed in parental and STEAP1 KO cells (Supplemental Figure 11A). No cytotoxic effects of naked PSMA scFv-Fc or STEAP1 scFv-Fc were observed in C4-2B or 22Rv1 cells (Supplemental Figure 11B). After ensuring antigen specificity, PSMA scFv-Fc was conjugated to tesirine (SG3249), a pyrrolobenzodiazepine dimer payload, resulting in PSMA-SG3249 ADC with a drug-to-antibody ratio (DAR) of 3. STEAP1 scFv-Fc was conjugated to the camptothecin analog deruxtecan (DXd), resulting in STEAP1-DXd with a DAR of 1.09. Enzymatically cleavable linkers were used in all 3 ADC designs (Supplemental Table 6).

B7-H3–seco-DUBA, PSMA-SG3249, and STEAP1-DXd demonstrated reasonable selective toxicity against cells expressing target antigens (Figure 6A). These ADCs were subsequently evaluated for their ability to activate an adaptive anti-apoptotic response in PC cells. LNCaP cells were exposed to each ADC and evaluated for the abundance of BCL-2 family anti-apoptotic proteins over a time course (1, 6, 12, 24, and 48 hours; Figure 6B). ADC concentrations were selected to activate the p53-dependent pathway, as indicated by elevated p53 protein levels at 12–48 hours after exposure, without yet driving the cells to apoptosis (evidenced by the absence of PARP1 cleavage; Figure 6B). While we did not observe an increase in BCL-2 protein in any treatment condition (MSKCC EF1 NEPC cell lysate used as a positive control), we found that ADC exposure induced an increase in BCL-XL, MCL-1, and BCL-W (Figure 6B). The most robust and consistent increase was observed in BCL-XL at 24–48 hours across all 3 ADCs. These findings further support the rationale for combining genotoxic ADCs with BCL-XL inhibitor A-1331852 to enhance their cytotoxic effects.

**Figure 7:**
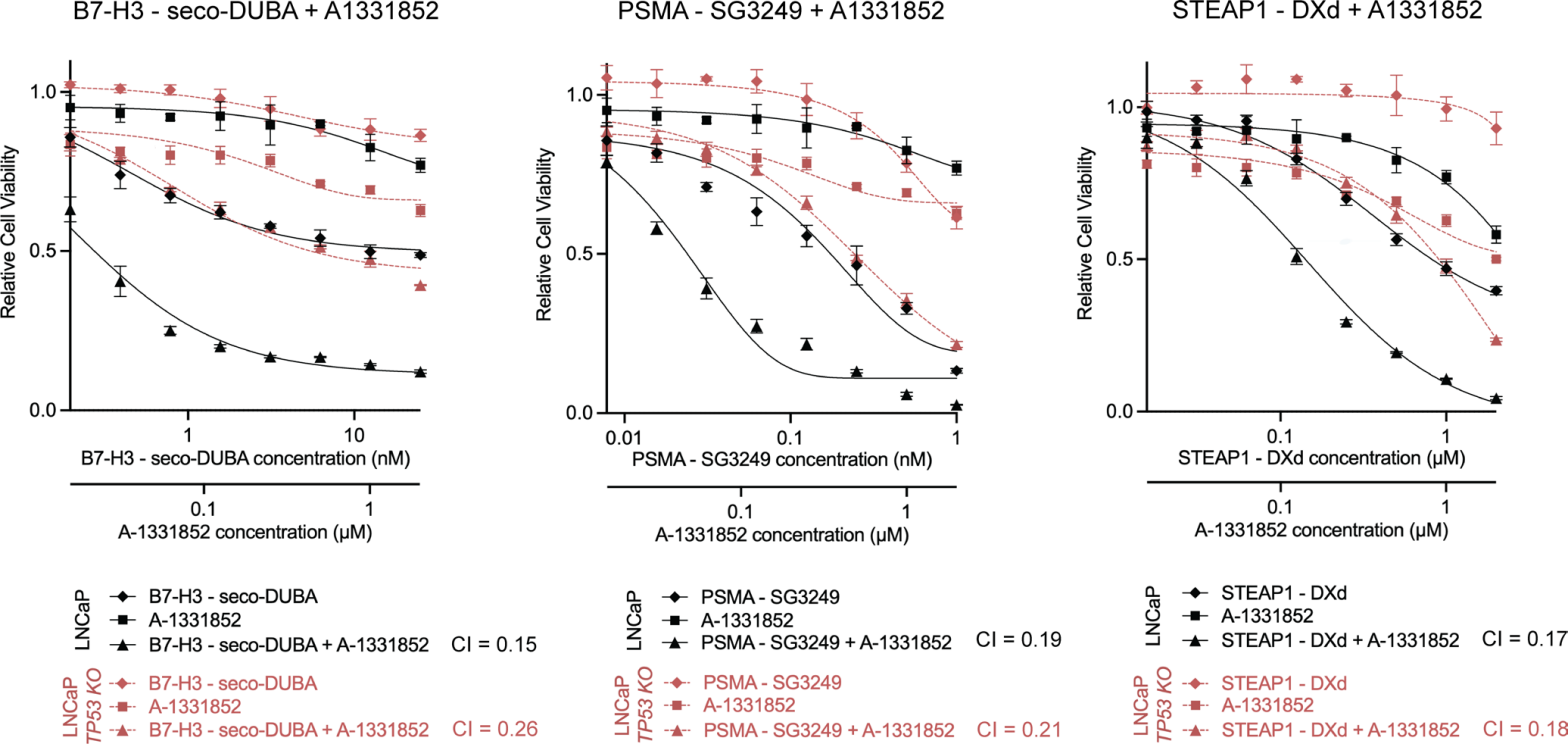
ADCs bearing genotoxic payloads synergize with A-1331852 in vitro. Synergistic interactions between genotoxic ADCs and BCL-XL inhibitor A-1331852 in *TP53* WT and *TP53-*KO LNCaP cells. Plots show mean ± SD (*n* = 3). CI, combination index.

B7-H3–seco-DUBA, PSMA-SG3249, and STEAP1-DXd were then applied to *TP53* WT and *TP53*-KO LNCaP cells, with and without A-1331852. Synergistic interactions between genotoxic ADCs and A-1331852 were observed in both cell models and in all ADC combinations tested (Figure 7); however, the responses were greater in p53-proficient cells. Because B7-H3 is expressed in NEPC and *RB1/TP53* co-loss has been associated with increased replication stress, we next evaluated whether this combination could induce synergy in an *RB1/TP53* double-knockout (DKO) LNCaP model (Supplemental Figure 12). In these DKO cells, we observed reduced sensitivity to both B7-H3–seco-DUBA and A-1331852 as single agents, as well as to the combination, consistent with the therapeutic resistance frequently associated with *RB1/TP53*-deficient PC (52). Although *RB1* loss has been linked to increased dependence on BCL-XL signaling (53), our data suggest that concurrent *TP53* loss may attenuate the response to this therapeutic strategy in the LNCaP context. Importantly, the LNCaP *RB1/TP53-DKO* model does not fully recapitulate the NE phenotype; therefore, these findings may not reflect the vulnerabilities of clinical NEPC. Consistent with this possibility, the established NEPC cell line MSKCC EF1 displayed sensitivity to A-1331852 (Figure 3B and Supplemental Figure 6), likely reflecting the distinct molecular landscape and apoptotic dependencies characteristic of NEPC.

To rule out potential effects of A-1331852 on ADC internalization and membrane trafficking, MGC018 was labeled with a pH-sensitive pHrodo dye and applied to C4-2B cells as a single agent or in combination with A-1331852 (Supplemental Figure 13). The intracellular ADC signal was monitored over time using fluorescence microscopy (Supplemental Figure 13A), with C4-2B B7-H3–KO cells serving as a negative control. We observed no substantial changes in signal intensity or the rates of ADC internalization regardless of the presence of 0.1 or 1 μM A-1331852 (Supplemental Figure 13, B and C). Furthermore, no MGC018 internalization was detected in the B7-H3–KO cells. These findings demonstrate that BCL-XL inhibition does not markedly affect ADC uptake or trafficking.

### Antitumor activity of MGC018 and A-13318562, alone or in combination, in p53-deficient and -proficient mCRPC.

The antitumor activity of B7-H3–seco-DUBA ADC (MGC018) and its combination with unconjugated A-1331852 in vivo was examined using C4-2B and C4-2B *TP53-*KO cell-derived xenograft (CDX) tumors established in male NSG mice. Mice were treated with vehicle, B7-H3–targeted ADC, A-1331852, or the combination of B7-H3–targeted ADC and A-1331852 for a period of 2 weeks. Tumor growth was monitored for an additional 2 weeks after the end of treatment. To assess the joint activity of A-1331852 and MGC018, we utilized a dosing strategy that produced incomplete tumor responses for both agents, thereby avoiding a response ceiling. For A-1331852, we administered 25 mg/kg (PO, 5 days/week), an established dose used for the combination with cytotoxic agents in preclinical xenograft models (54–56). For MGC018, we conducted a dose-finding study in C4-2B CDXs (Supplemental Figure 14) to identify a dose that achieved a consistent but incomplete tumor response. While literature reports antitumor efficacy for MGC018 across a range of 0.3–10 mg/kg (19), our analysis established 3 mg/kg (i.p., weekly) as the optimal dose for combination studies, ensuring the monotherapy provided sufficient room to observe enhanced tumor control in the combination group.

**Figure 8:**
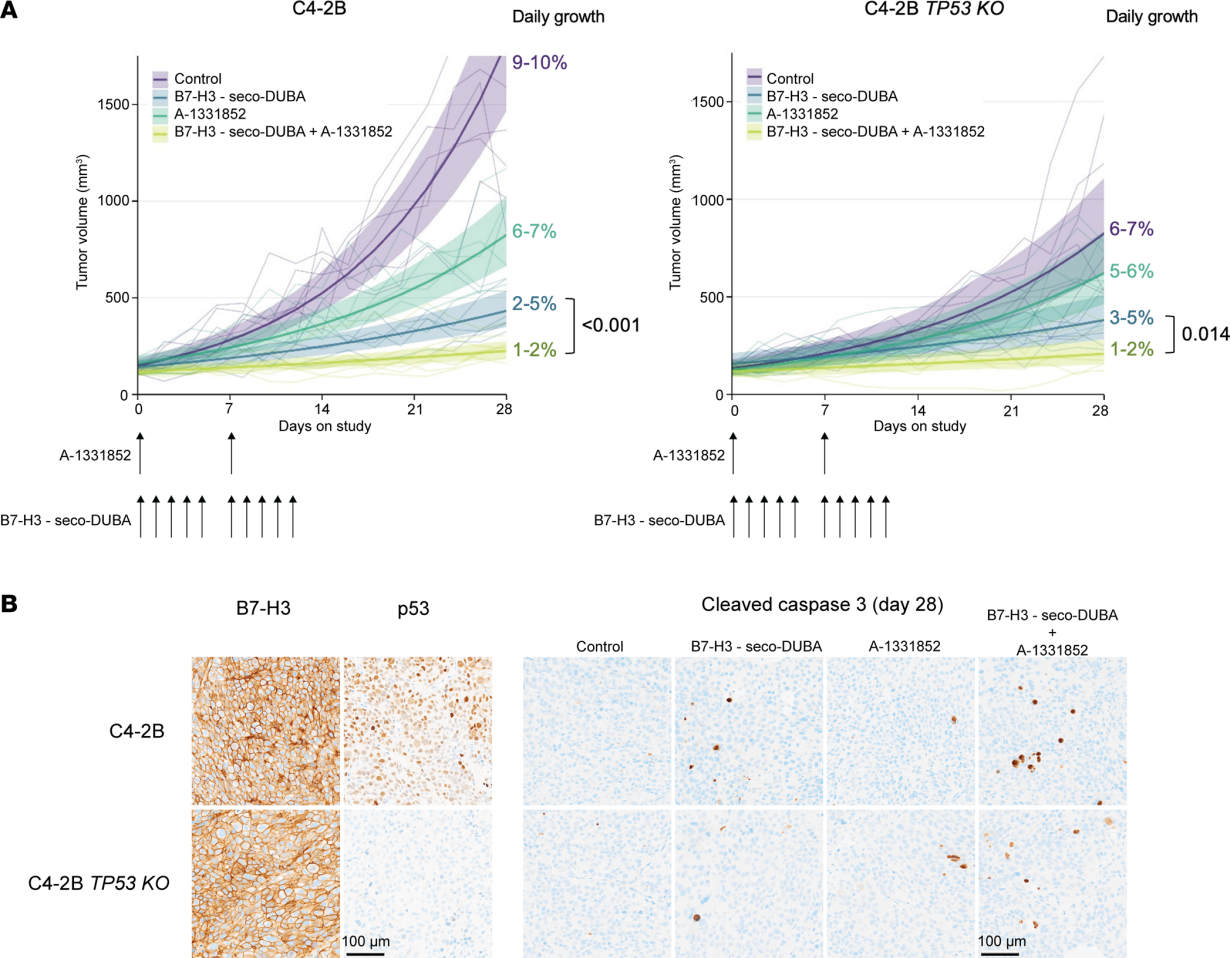
Combination B7-H3–seco-DUBA ADC with systemic A-1331852 inhibits the growth of p53-proficient and -deficient mCRPCs. (**A**) Volumetric changes of C4-2B and C4-2B *TP53-*KO xenograft tumors in NSG mice (*n* = 6–7) exposed to vehicle alone, B7-H3–seco-DUBA ADC (3 mg/kg i.p.), A-1331852 (25 mg/kg PO), and the combination of an ADC (3 mg/kg) with A-1331852 (25 mg/kg). Data represent mean ± SEM. (**B**) Immunohistochemical analysis of B7-H3 and p53, as well as cleaved caspase 3, in control and experimental groups at study termination. Scale bars: 100 �m.

Overall, we observed a greater and more rapid inhibition of tumor growth in response to MGC018 in p53-proficient tumors (Figure 8A and Supplemental Figure 15A). The addition of BCL-XL inhibitor significantly enhanced antitumor activity in combination with MGC018 in both C4-2B and C4-2B *TP53*-KO CDXs (Figure 8A and Supplemental Figure 15A). In the C4-2B experiment, tumor volume in the control group grew 9%–10% per day. The BCL-XL inhibitor A1331852 reduced the average daily growth rate to 6%–7%. MGC018 reduced the average daily growth rate to 2%–5%, while the combination of both drugs reduced growth to 1%–2%. In the C4-2B *TP53*-KO experiment, tumors were smaller, and the volume in the control group grew on average 6%–7% per day. The BCL-XL inhibitor A1331852 reduced the average daily growth rate to 5%–6%. MGC018 reduced the average daily growth rate to 3%–5%. The combination of both MGC018 and A-1331852 reduced the daily growth rate to 1%–2%. In C4-2B CDXs, adding A1331852 to MGC018 reduced the average daily growth rate by 2%–3% (95% CI, *P* < 0.001), while in the C4-2B *TP53*-KO model, it reduced the average daily growth rate by 1%–3% (95% CI, *P* = 0.014) (Supplemental Figure 15A).

To confirm target antigen expression and p53 status in the tumor tissues, samples were collected at days 14 and 28 of the experiment, and B7-H3 and p53 proteins were evaluated by IHC. We did not observe any differences in B7-H3 expression in C4-2B or C4-2B *TP53-*KO tumors treated with MGC018 (Figure 8B). As expected, p53 staining revealed nuclear p53 expression in C4-2B tumors but absence in C4-2B *TP53-*KO tumors (Figure 8B). Apoptosis induction was analyzed via IHC staining of the tumors harvested at study termination (Figure 8B). Moderate body weight loss was observed in animals treated with A-1331852, singly or combined with ADC during the first 2 weeks of the experiment (Supplemental Figure 15B), suggesting that the safety of this drug might be improved by using A-1331852 as an ADC payload. Importantly, the combination of MGC018 with A-1331852 did not accelerate weight loss (Supplemental Figure 15B).

We next investigated the potential systemic side effects associated with this treatment strategy. Prior studies have established that inhibition of BCL-XL leads to on-target hematologic toxicity, most notably thrombocytopenia, reflecting the dependence of platelets on BCL-XL for survival (57, 58). Thrombocytopenia has been reported as a dose-limiting toxicity in studies evaluating pharmacologic BCL-XL inhibition, including with the small-molecule inhibitor A-1331852 (55). To examine whether combination therapy with A-1331852 affects platelet counts, we conducted an additional small study (*n* = 3) in which animals were exposed to the same single-agent and combination treatment regimens described above. Platelet counts were analyzed 24 hours after the final A-1331852 dose (Supplemental Figure 15C). Consistent with the studies performed using C4-2B and C4-2B *TP53-*KO CDX models, animals treated with A-1331852 exhibited modest weight loss (Supplemental Figure 15C). The average platelet count was lower in the A-1331852–treated groups; however, within this small cohort, animals treated with A-1331852 alone or in combination with MGC018 did not develop acute thrombocytopenia (Supplemental Figure 15C). Furthermore, the combination of the ADC with A-1331852 did not exacerbate weight loss or reduce platelet counts compared with A-1331852 alone.

Collectively, these findings demonstrate that the addition of a BCL-XL inhibitor to an ADC bearing a DDD payload can improve tumor responses in both p53-proficient and -deficient PC. Systemic toxicities associated with unconjugated A-1331852 remain a potential safety concern, consistent with prior reports. Because unconjugated A-1331852 was utilized for proof-of-principle validation, an exhaustive safety profiling of this specific combination was not pursued. A rational strategy to mitigate systemic effects involves incorporating A-1331852 as an ADC payload in dual-ADC regimens. Additionally, our xenograft experiments were conducted in immunocompromised mice, which limits our ability to evaluate potential on-target toxicities of the ADCs in normal tissues. Future studies using humanized mouse models expressing the target antigen will help address these limitations. While the present study establishes a proof of concept for combining synergistic ADC payloads, further work is required to evaluate the therapeutic efficacy and safety of ADCs incorporating DDDs and BCL-XL inhibitors, as well as the potential benefit of combination strategies involving these agents.

## Discussion

The development and translation of effective precision therapies for mCRPC are necessary to alter the course of this highly aggressive disease. Tumor responses to anticancer treatments are often improved by combining cytotoxic agents that operate through diverse MoAs, and the tolerability of these agents can be enhanced with the use of more effective tumor delivery systems. ADCs offer a unique platform for the development of custom large molecules that target single or multiple tumor-associated antigens and deliver single or multiple cytotoxins (payloads) to the tumor site. Rationally designed combinatorial ADC-based regimens, including simultaneous or sequential ADC coadministration, ADCs combined with systemic agents, and dual-payload ADCs, have the potential to make a clinical impact on the management of mCRPCs.

Most ADC targets are tumor-associated antigens with high abundance in cancer cells and limited normal tissue expression (4–8, 59). When ADC affects healthy cells expressing low levels of target antigen, systemic toxicity may arise. Such on-target ADC effects can potentially be reduced by the use of 2 ADCs targeting different surface antigens, each administered at lower doses, as fewer normal tissues would coexpress 2 tumor-associated targets. In this work, we ascertained the coexpression of PC surface antigens in malignant and in benign tissues to strategically select tumor-restricted combinations for mCRPC. mCRPC is a phenotypically heterogeneous disease with tumor subtypes marked by distinct transcriptional programs (21, 22) and cell surface antigen expression (11, 16, 60–62). Our analysis of coexpression patterns of PC associated surface antigens across 4 mCRPC molecular phenotypes demonstrated that B7-H3(*CD276*), PSMA(*FOLH1*), and STEAP1 were frequently coexpressed in the same cells in the tumors with sustained AR signaling. Importantly, coexpression of either explored antigen pair is limited in normal tissues. Thus, our data provide rationale for the use of ADCs cotargeting B7-H3 and STEAP1, STEAP1 and PSMA, and B7-H3 and PSMA in AR-active tumors, which represent about 80% of mCRPCs (21, 22). A major challenge for multiantigen-targeted strategies is the spatial and temporal heterogeneity of target expression across metastatic sites. While PET-based tracking for B7-H3 and STEAP1 is not yet routine, emerging liquid biopsy platforms offer a promising, minimally invasive tool to supplement baseline IHC for real-time patient stratification. Recent profiling of circulating tumor cells and tumor-derived extracellular vesicles in mCRPC cohorts demonstrates that PSMA, B7-H3, and STEAP1 expression can be tracked dynamically over time from peripheral blood (63, 64). Integrating these liquid biomarkers as companion diagnostics alongside conventional tissue biopsies could facilitate longitudinal monitoring of antigen landscapes, helping optimize patient selection while bypassing the limitations of serial regional tissue biopsies.

Purposeful selection of ADC payloads and incorporation of complementary drugs provide another opportunity to improve treatment efficacy. Most ADCs that have been investigated in PC clinical trials employed microtubule disrupting monomethyl auristatin E (MMAE) as a payload, and none of these ADCs demonstrated a satisfactory therapeutic window in mCRPC patients (6). It remains unclear whether MMAE is an optimal cytotoxic agent to control the growth of mCRPCs that were previously exposed to taxane therapy and could have developed resistance mechanisms. Our screening of 23 small molecules from 3 major ADC payload classes determined that AR-active mCRPC cells had greater average responses to DNA-damaging payloads compared with responses to microtubule-targeting drugs. We also found that an NEPC model had remarkable responses to several innovative payloads, including the BCL-XL inhibitor A-1331852. Further exploration of this result in a larger panel of NEPC models might enable the development of A-1331852–bearing ADCs for this mCRPC subtype that currently has limited treatment options beyond platinum-based chemotherapy.

Through the screening of 78 payload combinations, we identified synergism between DDDs and A-1331852. We found that exposure to this payload combination initiates the intrinsic p53-dependent apoptosis pathway and that BCL-XL (*BCL2L1*) expression and p53 activity associate with maximal cytotoxicity. Although synergism was observed in both p53-deficient and -proficient PC models, isolated genetic *TP53*-KO reduced cell sensitivity to 5 DDDs and 3 DDD-bearing ADCs, as well as their combinations with A-1331852. In a prior study assessing the efficacy of a B7-H3 ADC with a pyrrolobenzodiazepine payload in 26 treatment-resistant PC PDXs, WT *TP53* status predicted nonresponsiveness (12). However, *TP53* loss in mCRPC is generally not an isolated event, and other genomic and phenotypic features may contribute to ADC efficacy and synergism. Further investigation into p53 status as a biomarker of response to genotoxic ADCs and the influence of other genomic alterations, including *BRCA2* and *RB1* mutations, is required to direct a precision medicine–based approach to ADC therapy.

We confirmed that the BCL-XL inhibitor A-1331852 enhances antitumor activity of genotoxic payloads by combining B7-H3–seco-DUBA ADC with unconjugated A-1331852 using in vivo C4-2B and C4-2B *TP53-*KO tumor models. These findings have implications for the clinical development of next-generation ADC combinations. Synergism between genotoxic payloads and A-1331852 could be exploited by the development of dual-payload ADCs for the treatment of AR-active and NE mCRPCs. Tumor specificity of these drugs could be further improved by combining 2 ADCs bearing DDD and A-1331852 payloads. Although concurrent administration of 2 synergistic ADCs may substantially reduce off-tumor toxicity, it will be crucial to assess clinical pharmacokinetics and tumor penetration of 2 large molecules in this scenario. Inherent structural variability of ADC constructs, including differences in DAR, linker chemistry, and binding affinity, may act as an additional confounding variable. Future studies must rigorously control for these factors to design optimal combinations.

Our data also suggest that PC cells may exhibit compensatory expression of anti-apoptotic BCL-2 family proteins, including BCL-XL and MCL-1 (Figure 6B), which could influence therapeutic responses. While our findings demonstrate the efficacy of BCL-XL–targeting strategies in mCRPC, the selection of A-1331852 in this study was guided by an empirical, unbiased screen of ADC-compatible payloads available at the time (Figure 3A). Importantly, the landscape of proapoptotic ADC payloads has since expanded. Recent advances have enabled the conjugation of MCL-1–targeting scaffolds, including S63845 (65). Although MCL-1 inhibitors were not included in our initial screening library, they represent promising opportunities for therapeutic diversification. Future studies should evaluate these agents, particularly in combination with DDD-based ADCs, to determine whether targeting complementary apoptotic pathways can overcome potential resistance mechanisms. Additionally, given the functional redundancy within the BCL-2 family (66), compensatory reliance on BCL-2 or MCL-1 can drive resistance following BCL-XL inhibition. Future studies should characterize these feedback dynamics, as targeting these complementary apoptotic pathways could expose secondary vulnerabilities and prevent treatment evasion when paired with genotoxic ADCs.

In summary, we have introduced the concept of cotargeting mCRPCs with ADCs bearing synergistic payloads. We characterized the coexpression of clinically relevant cell surface targets B7-H3, PSMA, and STEAP1 in mCRPC and identified synergism between genotoxic payloads and the BCL-XL inhibitor payload A-1331852 and others that are undergoing further validation. These findings establish the initial foundation for future clinical development of effective ADC combinations for mCRPC. Successful clinical translation of this combination will require patient stratification based on antigen coexpression, necessitating future studies to define optimal target density thresholds. Integrating molecular profiling for *TP53* status and other genomic alterations into early-phase clinical studies will be essential to refine these biomarkers, predict patient responses, and optimize treatment selection in mCRPC.

## Methods

### Sex as a biological variable.

The research described in this study utilized only male mice and male human tumor samples. This approach was taken because PC is a male-specific disease.

### Cell lines and materials.

22Rv1 (RRID:CVCL_1045), LNCaP (RRID:CVCL_0395), C4-2B (RRID:CVCL_4784), VCaP (RRID:CVCL_2235), and 293T (RRID:CVCL_0063) cell lines were purchased from the ATCC. Cells were maintained in a 37°C incubator with 5% CO_2_ and grown in medium supplemented with FBS and other additives as recommended by ATCC. LuCaP cell lines were generated by resecting the tumor implant, dissociating cells by enzymatic digestion, and plating cells in DMEM medium with 10% FBS or with various additives used in organoid medium (67). MSKCC EF1 (derived from the organoid line MSKCC-CaP4) (60) were maintained in RPMI medium supplemented with 10% FBS, 100 U/mL penicillin, 100 μg/mL streptomycin, and 4 mmol/L GlutaMAX. FreeStyle 293-F cells (RRID:CVCL_D603) were purchased from Thermo Fisher Scientific and maintained in serum-free FreeStyle 293 Expression Medium on an orbital shaker in 37°C incubator with a humidified atmosphere of 8% CO_2_. Cell lines were cultured no more than 3 weeks after thawing prior to use in described experiments. Cell lines underwent DNA fingerprint (short terminal repeat) confirmation and routine mycoplasma testing (R&D Systems, CUL001B) via Fred Hutchinson Cancer Center Research Cell Bank Services. MGC018, vobramitamab duocarmazine, vobra duo was provided by MacroGenics.

### Immunoblotting.

Lysates were prepared in RIPA buffer (Millipore) supplemented with Pierce protein inhibitor cocktail (Thermo Fisher Scientific), pelleted to remove debris, then quantified by BCA assay (Thermo Fisher Scientific). Extracts were fractionated by SDS-PAGE and transferred to a PVDF membrane. Membranes were blocked with 5% nonfat milk in TBST (TBS + 1% Tween 20) for 1 hour while shaking, then incubated with primary antibodies at 4°C for 16 hours. Primary antibodies targeting AR (Abcam, catalog ab133273, RRID:AB_11156085, 1:5,000), p53 (Santa Cruz Biotechnology, catalog sc-126, RRID:AB_628082, 1:500), BCL-2 (Santa Cruz Biotechnology, catalog sc-7382, RRID:AB_626736, 1:500), BCL-XL (Cell Signaling Technology, catalog 2764, RRID:AB_2228008, 1:1,000), MCL-1 (Cell Signaling Technology, catalog 94296, RRID:AB_2722740, 1:1,000), BCL-W (Cell Signaling Technology, catalog 2724, RRID:AB_10691557, 1:500), RB (BD Biosciences, catalog 554136, RRID:AB_395259, 1:1,000), PARP1 (Thermo Fisher Scientific, catalog 436400, RRID:AB_2532215, 1:1,000), cleaved caspase-3 (Cell Signaling Technology, catalog 9661, RRID:AB_2341188, 1:1,000), B7-H3 (Sigma-Aldrich, SAB5500011, 1:5,000), PSMA (Agilent, catalog M3620, RRID:AB_2106450, 1:1,000), STEAP1 (Cell Signaling Technology, catalog 88677, RRID:AB_2800128, 1:1,000), and GAPDH (Santa Cruz Biotechnology, catalog sc-47724, RRID:AB_627678, 1:5,000) were used. Membranes were washed 3 times with TBST and incubated with HRP-conjugated anti-mouse (Agilent, catalog P0447, RRID:AB_2617137) or anti-rabbit (Leica Biosystems, catalog PV6119, RRID:AB_1307590) secondary antibody for 1 hour at room temperature. Blots were washed 3 times with TBST and developed with Immobilon Western Chemiluminescent HRP Substrate (Millipore). Blot images were acquired with a ChemiDoc MP Imaging System (Bio-Rad).

### Payload screening.

The 23 compounds used in the screen are described in Supplemental Table 1. Staurosporine (APExBIO, A8192) at 1 μM final concentration was used as positive control. All drugs were reconstituted in DMSO and manually arrayed in a 384-well master plate at concentrations 500-fold greater than final concentration. Drug screening was performed at Fred Hutchinson Genomics and Bioinformatics Shared Resource High Throughput Screening lab. Submicroliter aliquots of compounds were applied to 384-well culture plates (Corning, 3570BC) using a Beckman Coulter Biomek i7 liquid handling instrument fitted with a V&P Scientific 384 Pin Tool applicator. 1–10 × 10^3^ cells in suspension were applied to culture plates containing drug aliquots with a BioTek MicroFlo Select dispense instrument to a final volume of 30 μL and incubated for 3 days. To assess cell viability, 30 μL of CellTiter Glo 2.0 reagent (Promega) was applied to culture plates with a BioTek MicroFlo Select dispense instrument. Plates were incubated for 20 minutes on a platform shaker and then read using a Perkin-Elmer EnVision 2104 multilabel reader.

### Drug dose-response curves.

Cells were seeded at 5–20 × 10^3^ cells (100 μL) per well in 96-well, tissue culture–treated, clear bottom, white plates (Corning). Cells were treated with serial dilutions in replicates of 3, at 37°C for 72 hours for free toxin dose response or for 96 hours for ADC dose response. Cell viability was determined using the CellTiter-Glo 2.0 Assay (Promega).

### Generation of B7-H3–, PSMA-, and STEAP1-KO cell lines.

B7-H3/*CD276-*, PSMA/*FOLH1-*, and STEAP1-KO cells were generated by transient transfection of cells with a pool of PX458 plasmids (Addgene, 48138), each expressing 1 sgRNA targeting sequence. The following were used for B7-H3/*CD276* sgRNAs: 5′-GTGGTCACGTTGCCAGTCAG-3′, 5′-CTGGTGCACAGTTTCACCGA-3′, and 5′-CACAGGGCAACGCATCCCTG-3′; for PSMA/*FOLH1* sgRNAs: 5′-TTATAGGCGTGGAATTGCAG-3′, 5′-GGAGAGAAAGCACTGAAAGG-3′, 5′-GGTACACAACCTAACAAAAG-3′, and 5′-CTGTTGTTCATGAAATTGTG-3′; and for STEAP1 sgRNAs: 5′-ATAGTCTGTCTTACCCAATG-3′, 5′-CCTTTGTAGCATAAGGACAC-3′, 5′-ATCCACTTATCCAACCAATG-3′, and 5′-CATCAACAAAGTCTTGCCAA-3′. 48–72 hours after transfection, GFP^+^ cells were singly sorted on a Sony SH800 Cell Sorter into a 96-well plate and clonally expanded.

### Generation of TP53-KO lines.

sgRNA (5′-CCCCGGACGATATTGAACAA-3′) was first cloned into the plentiCRISPRv2-blast vector (RRID:Addgene_98293). Lentivirus carrying the construct was then used to transduce C4-2B and LuCaP 189.4 cells, which were subjected to blasticidin selection for 5–7 days. Subsequently, C4-2B cells were plated at a single-cell density to enable clonal expansion. The resulting colonies were expanded and analyzed by Western blot. Genomic junctions were characterized by amplifying the sgRNA-targeted regions with sequence-specific primers, followed by Sanger sequencing. LNCaP *TP53*-KO and LNCaP *RB1*/*TP53*-KO (DKO) cell lines were described in a prior study (52).

### PSMA and STEAP1 scFv-Fc expression plasmids.

gBlocks (IDT) encoding J591 or DSTP3086S scFv (VL-[G4S]3-VH) were cloned into the SfiI site of TGEX-SCBlue mammalian expression vector (Antibody Design Labs) using HiFi DNA Assembly (New England Biolabs). Resulting TGEX-FOLH1 and TGEX-STEAP1 constructs were analyzed by Sanger sequencing.

### Production of PSMA and STEAP1 scFv-Fc antibodies.

Protein sequences of scFv-Fc constructs are listed in Supplemental Table 7. Recombinant scFv-Fc antibodies were produced using the FreeStyle 293 Expression system (Thermo Fisher Scientific). Briefly, 293-F cells were transfected with TGEX-FOLH1 or TGEX-STEAP1 expression vectors using FreeStyle MAX Reagent and grown in serum-free FreeStyle 293 Expression medium for 7 days. Conditioned media was clarified by centrifugation and filter sterilized using Millipore 0.22 μm SteriCup vacuum filtration units. Clarified supernatant was passed through a Cytiva HiTrap Protein G HP column, and then bound antibodies were eluted with glycine-HCl buffer (0.1 M glycine, 0.1 M NaCl, pH 2.7), neutralized, and dialyzed against PBS using Slide-A-Lyzer dialysis cassettes (Thermo Fisher Scientific). After dialysis, antibodies were concentrated on 10K MWCO Amicon Ultra-15 centrifugal filter units. Antibody preparations were characterized by SDS-PAGE analysis; protein concentrations were measured by bicinchoninic acid assay (Thermo Fisher Scientific).

### Antibody conjugation.

Conjugation of PSMA scFv-Fc with tesirine (*Mal-PEG8-Val-Ala-PABC*-SG3249) or DXd (*MC-GGFG*-DXd) linker-payload (LP) was performed by NJ Bio, Inc. The antibody, 5 mg/mL in 1× PBS, was reacted by 10-fold molar excess of Tris(2-carboxyethyl)phosphine. The reaction solution was incubated at 37°C for 90 minutes. Then, 10% (v/v) dimethylacetamide was added to the solution, followed by 15-fold molar excess of the LP, incubated at room temperature for 3 hours. The LP was prepared at 10 mM in dimethylacetamide. To remove the excess LP and aggregation, size-exclusion chromatography was applied. A Hiload 16/600 Superdex 200 column was equilibrated by 1× PBS buffer, prior to injecting the reaction solution. After size-exclusion chromatography purification, the ADC was filtered using a 0.22 μm polyethersulfone filter and stored at 4°C. The DAR was calculated via liquid chromatography–mass spectrometry (LC-MS) using 0.5 μg of reduced material injected over reverse-phase LC-MS (1,000 Å, 8 μm, 2.1 × 50 mm).

Conjugation of STEAP1 scFv-Fc with DXd LP was performed using an Antibody Deruxtecan Conjugation Kit (CellMosaic, DCM11431) according to the manufacturer’s protocol. DAR was calculated via hydrophobic interaction chromatography analysis (CellMosaic).

### Flow cytometry.

Parental C4-2B and 22Rv1, as well as PSMA-KO or STEAP1-KO cell lines, were dissociated nonenzymatically with Versene-EDTA into single-cell suspensions. 0.5 million cells were washed thrice with monoclonal antibody wash (MW; 1× PBS + 0.1% FBS + 0.1% NaN_3_) and resuspended in 200 μL of 5 μg/mL of PSMA scFv-Fc or STEAP1 scFv-Fc and incubated at 4°C on ice for 1 hour. Cells were then washed with MW, incubated with PE anti-human secondary antibody (BioLegend, catalog 410707, RRID:AB_2565785) at 4°C on ice for 30 minutes, washed with MW, acquired on a SH800 (Sony), and analyzed with FlowJo (RRID:SCR_008520).

### B7-H3–seco-DUBA (MGC018) lysosome trafficking.

To monitor ADC internalization and lysosomal trafficking, ADC was linked with pH-sensitive Zenon pHrodo iFL Red-labeled Fab fragment. C4-2B and C4-2B B7-H3–KO cells were transduced with H2B-GFP (construct, RRID:Addgene 25999). 5,000 cells were seeded in 25 �L per well in a 96-well half-area plate (Greiner, 675096). The next day, DMSO or A-1331852 was added to cells (5 �L of 10× DMSO, 0.1 or 1 �M A-1331852), and the plate was put at 4°C while preparing antibody. MGC018 + phRodo label was prepared (3:1 molar ratio dye to ADC), then 25 �L of labeled ADC was added to each well (10 nM ADC final concentration in 55 �L total volume). Cells were incubated for 30 minutes at 4°C, then placed in a BioTek Cytation 5 for live-cell imaging. Wells were read in bright-field, GFP, and rhodamine channels every 2 hours, starting at 1 hour after ADC addition, up to 49 hours. Additional images were taken at 72 and 96 hours. A ×20 objective, wide-field fluorescence microscope and 5 × 4 tiling per well (1.3 × 1.1 mm area) were used for imaging. Images were processed with cellpose for cell segmentation using the CPSAM deep learning model (68), and cell fluorescence was measured in ImageJ (NIH). There were 395–554 cells per treatment analyzed at the initial analyzed time point (3 hours). Fluorescence signal (mean per cell) was normalized versus the 3-hour time point.

### Animal studies.

Animal care and studies were performed in accordance with an approved Fred Hutchinson Cancer Center Institutional Animal Care and Use Committee protocol and Comparative Medicine regulations. 2 × 10^6^ C4-2B or C4-2B *TP53-*KO cells were suspended in 100 μL of cold 50% Matrigel (Corning)/PBS and implanted by injection subcutaneously into 8- to 10-week-old male NSG mice (NOD-SCID-IL2Rγ-null, RRID:BCBC_4142). Mice were originally obtained from The Jackson Laboratory and were bred internally by the Specialized Mouse Services Core of the Cooperative Center for Excellence in Hematology (CCEH) at Fred Hutchinson Cancer Center. Animals were assigned to experimental groups using simple randomization, enrolled into experiments when tumors reached 100 mm^3^, and treated by intraperitoneal injection and oral gavage at the frequency and with the doses described. MGC018 was formulated in PBS and administered intraperitoneally (100 μL, at 3 mg/kg) once a week for 2 weeks. A-1331852 was formulated in 2.5% DMSO, 10% ethanol, 27.5% PEG 300, and 60% Phosal 50 PG. A-1331852 doses (100 μL, at 25 mg/kg) were administered by oral gavage once a day, with a 5 days on/2 days off regimen for 2 weeks. Mice were monitored every other day for tumor growth, weight, and body condition score. All enrolled animals finished the study, i.e., there was no attrition. In the toxicity study, adult mice received 2 doses of MGC018 and 10 doses of A-1331852. 24 hours following the final dose, blood was collected via terminal cardiac puncture under deep surgical anesthesia. Whole blood was collected in EDTA-coated tubes and submitted for complete blood count analysis using an Element HT5 hematology analyzer (Heska Corporation).

### mIF staining.

Primary and secondary antibodies used for mIF are listed in Supplemental Table 2.

FFPE tissues were baked for 1 hour at 65°C. The slides were then dewaxed and stained on a Leica BOND RX stainer using Leica Bond reagents for dewaxing (Dewax Solution), antigen retrieval/antibody stripping (Epitope Retrieval Solution 2) and rinsing after each step (Bond Wash Solution). Antigen retrieval and antibody stripping steps were performed at 100°C, with all other steps at ambient temperature. Endogenous peroxidase was blocked with 3% H_2_O_2_ for 5 minutes followed by protein blocking with TCT buffer (0.05 M Tris, 0.15 M NaCl, 0.25% casein, 0.1% Tween 20, 0.05% ProClin300, pH 7.6) for 10 minutes. The first primary antibody (position 1) was applied for 60 minutes, followed by the secondary antibody (Akoyo Biosciences, 1× Opal Anti-Ms + Rb HRP Polymer) application for 20 minutes, then the application of the tertiary TSA amplification reagent (Akoya Biosciences, OPAL fluor) for 20 minutes. A high-stringency wash was performed after the secondary and tertiary applications using a high-salt TBST solution (0.05 M Tris, 0.3 M NaCl, 0.1% Tween20, pH 7.2–7.6). The primary and secondary antibodies were stripped with retrieval solution for 20 minutes before repeating the process with the second primary antibody (position 2), starting with a new application of 3% H_2_O_2_. The stripping step was not performed after the final position. Slides were removed from the stainer and stained with a 5 μg/mL concentration of DAPI (Sigma-Aldrich, D8417) for 5 minutes, rinsed, and placed on coverslips with Prolong Gold Antifade reagent (Invitrogen/Life Technologies, P36930). After curing at room temperature, whole-slide images were acquired on the Vectra Polaris Quantitative Pathology Imaging System (Akoya Biosciences). The entire tissue was selected for imaging using Phenochart, and multispectral image tiles were acquired using the Polaris system. Images were spectrally unmixed using Phenoptics inForm software and exported as multi-image TIF files. The TMA slides were visualized with HALO (Indica Labs). H-scores were generated from the mIF data using the CytoNuclear LCv2.0.6 module and HALO software. Individual cells were classified as having negative, weak, moderate, or strong staining and assigned intensity scores. The intensity scores were then multiplied by the percentage of stained cells for a range of 0–300. The triplicate scores were averaged to generate an averaged H-score for each site. To assess total percentage of positive or double-positive cells in the tumor, core percentages of cells with weak, moderate, or strong staining were combined.

### Chromogenic IHC.

IHC was conducted on the Ventana Discover Ultra automated platform (Ventana Medical Systems) automated platform. Sections were cut at a thickness of 4 μm from FFPE PDX tumors and mounted onto positively charged slides. Prior to onboard deparaffinization and staining, slides were incubated at 65°C for 1 hour to remove excess paraffin. Slides were then loaded onto the machine and incubated in Discovery wash buffer (Roche Diagnostics, 950-510), and heat-induced epitope retrieval was conducted in Discovery CC1 solution (Roche Diagnostics, 950-224). Primary antibodies B7-H3 (Sigma-Aldrich, SAB5500011, 1:500), P53 (Nolan lab, Stanford catalog 453M, RRID:AB_2864403, 1:50), and cleaved caspase-3 (Cell Signaling Technology, catalog 9664, RRID:AB_2070042, 1:1,000) were diluted in Ventana antibody diluent with casein (Roche Diagnostics, 760-219) and applied to the sections. The sections probed for P53 were washed and incubated with rabbit anti-mouse IgGs (Abcam, catalog ab46540, RRID:AB_2614925, 1:200). DISCOVERY anti-rabbit HQ secondary antibody (Roche Diagnostics, catalog 760-4815, RRID:AB_2811171) was applied to all slides, followed by the application of DISCOVERY anti-HQ HRP enzyme conjugate (Roche Diagnostics, 760-4820). The antibody-enzyme conjugate complex was visualized with ChromoMap DAB (Roche Diagnostics, 760-159). The sections were counterstained with Hematoxylin II (Roche Diagnostics, 790-2208) and Bluing Reagent (Roche Diagnostics, 760-2037). Slides were scanned at ×40 magnification on the Ventana DP 200 slide scanner (Roche Diagnostics).

### Gene expression and genomic analysis.

Published data of bulk flash-frozen needle biopsies from the SU2C-IDT/PCF cohort, bulk tumors from the UW mCRPC cohort, and LuCaP PDX tumors were sequenced and aligned as described previously (16). In brief, sequencing reads were mapped to the hg38 human genome using STAR v2.7.3a (RRID:SCR_004463). PDX data were also aligned to the mm10 mouse genome. All subsequent analyses were performed in R. PDX sequencing reads derived from mouse were removed using XenofilteR (https://github.com/PeeperLab/XenofilteR; commit ID 936c4a8). Gene-level abundance was quantitated using GenomicAlignments and transformed to log_2_ transcripts per million. Previously published *TP53* mutation status was determined as described by Frank et al. (69). Mutation status from samples with purity < 0.2 was omitted. Gene expression values shown in violin plots were compared using 2-sided Wilcoxon’s rank tests with Benjamini-Hochberg multiple-testing correction. Kaplan-Meier curves were estimated using the survival survfit function in R and plotted using survminer with overall survival from the time of biopsy, time on AR signaling inhibitor therapy, and time on docetaxel. Tumors were stratified by the 50th percentile (median) of expression values. The log-rank test was used to test for differences between survival curves.

### Statistics.

For each target antigen PSMA, STEAP1, and B7-H3, median differences in average H-scores between pairs of phenotypes were evaluated using Wilcoxon-Mann-Whitney rank-sum tests using Holm’s method to adjust for multiple comparisons using the wilcox_test function from the rstatix package (RRID:SCR_021240) in the R programming language (RRID:SCR_001905). This method was also used to evaluate median differences in relative viability between payload classes within each cell line. Intrapatient and intratumoral heterogeneity indices were estimated via bootstrapping from the same patient or tumor and evaluating whether pairs of samples had discordant positivity (i.e., 1 sample < 20% and 1 sample ≥ 20%). Longitudinal tumor volume measurements (on the logarithmic scale) were analyzed by fitting a linear model with random intercepts for independent animals and fixed effects for day, treatment group, and their interaction using the lmer function from the lmerTest package (RRID:SCR_015656); 2-sided *P* values for different slopes were used to determine effects of treatment on average daily tumor growth rates.

### Study approval.

The study was approved by the University of Washington (IRB 2341) and Fred Hutchinson Cancer Center (IRB 20257) Institutional Review Boards. All participating men provided written informed consent for a rapid research autopsy and tissue procurement. Animal experiments were carried out in accordance with the Fred Hutchinson Cancer Center’s approved protocol IR 1618.

### Data availability.

The UW mCRPC and LuCaP PDX RNA-seq data used in this study are available in the GEO repository (RRID:SCR_005012) under accession numbers GSE147250 and GSE199596. SU2C-IDT/PCF RNA-seq data are available in the cBioPortal (prad_su2c_2019) and dbGaP (phs000915.v2.p2.). Analytic code can be requested from the corresponding author. All experimental data on the single-case level are provided in the Supporting Data Values file.

## Author contributions

GS: conceptualization and design; acquisition, analysis, and interpretation of data; drafting and revision of the manuscript. SBF: conceptualization; acquisition, analysis, and interpretation of data; manuscript revision. RD, CDD, TA, and JM: data acquisition. WH: model development, manuscript drafting. IC: data analysis, manuscript drafting and revision. RG: data analysis, manuscript drafting and revision. CM: data acquisition, resources, manuscript revision. MCH: analysis and interpretation of data, manuscript revision. PSN: design of the research, data analysis, model supply, manuscript revision. JKL: conceptualization and design, resources, supervision, manuscript drafting and revision. All authors read and approved the final manuscript.

## Conflict of interest

PSN has served as a paid advisor to Genentech, AstraZeneca, Pfizer, and Janssen and received research support from Janssen. CM has received funds from Genentech and Novartis.

## Funding support

This work is the result of NIH funding, in whole or in part, and is subject to the NIH Public Access Policy. Through acceptance of this federal funding, the NIH has been given a right to make the work publicly available in PubMed Central.

- P30CA015704, P50CA097186, R50CA221836, R01CA266452, and R50CA274336 from the National Cancer Institute.
- W81XWH-14-2-0183, PC230582, and PC230420 from the Department of Defense Prostate Cancer Research Program and Congressionally Directed Medical Research Programs.
- The Prostate Cancer Foundation.
- Institute for Prostate Cancer Research.

## Supplementary Material

Supplemental data

Unedited blot and gel images

Supplemental table 3

Supplemental table 4

Supporting data values

## Version 1 06/25/2026

In-Press Preview

## Version 2 08/03/2026

Electronic publication
