# Supplementary material for: Genotoxic antibody-drug conjugates combined with BCL-XL inhibitors enhance therapeutic efficacy in metastatic castration-resistant prostate cancer
Source: J Clin Invest. 2026 Jun 23;136(15):e200438. doi: 10.1172/JCI200438 (PMC13430021; doi:10.1172/JCI200438)
Supplement: Supplemental data [file jci-136-200438-s068.pdf]

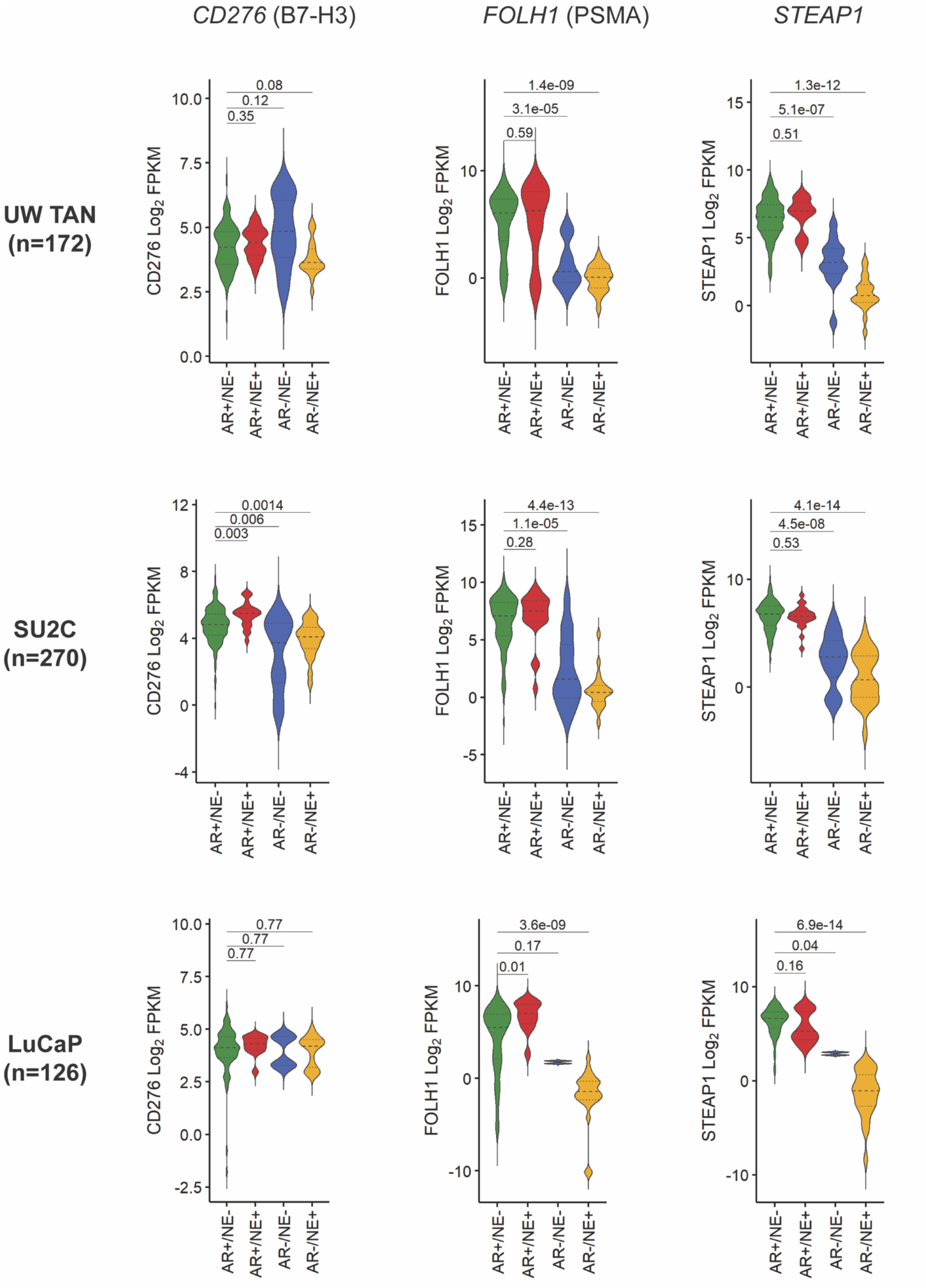

CD276 (B7-H3)
FOLH1 (PSMA)
STEAP1
UW TAN (n=172)
SU2C (n=270)
LuCaP (n=126)
CD276 Log2 FPKM
FOLH1 Log2 FPKM
STEAP1 Log2 FPKM
AR+/NE-
AR+/NE+
AR-/NE-
AR-/NE+
0.08
0.12
0.35
1.4e-09
3.1e-05
0.59
1.3e-12
5.1e-07
0.51
0.0014
0.006
0.003
4.4e-13
1.1e-05
0.28
4.1e-14
4.5e-08
0.53
0.77
0.77
0.77
3.6e-09
0.17
0.01
6.9e-14
0.04
0.16

Figure S1. ***CD276*** **(B7-H3),** ***FOLH1*** **(PSMA), and** ***STEAP1*** **expression across mCRPC molecular subtypes.**

Violin plots show *CD276*, *FOLH1*, and *STEAP1* transcript levels in AR+/NE-(green), AR+/NE+(red), AR-/NE-(blue), and AR-/NE+(yellow) tumors from UW TAN, SU2C, and LuCaP cohorts. Results are expressed as log2 fragments per kilobase of transcript per million mapped reads (FPKM). The groups were compared using two-sided Wilcoxon rank tests with Benjamini-Hochberg multiple-testing correction.

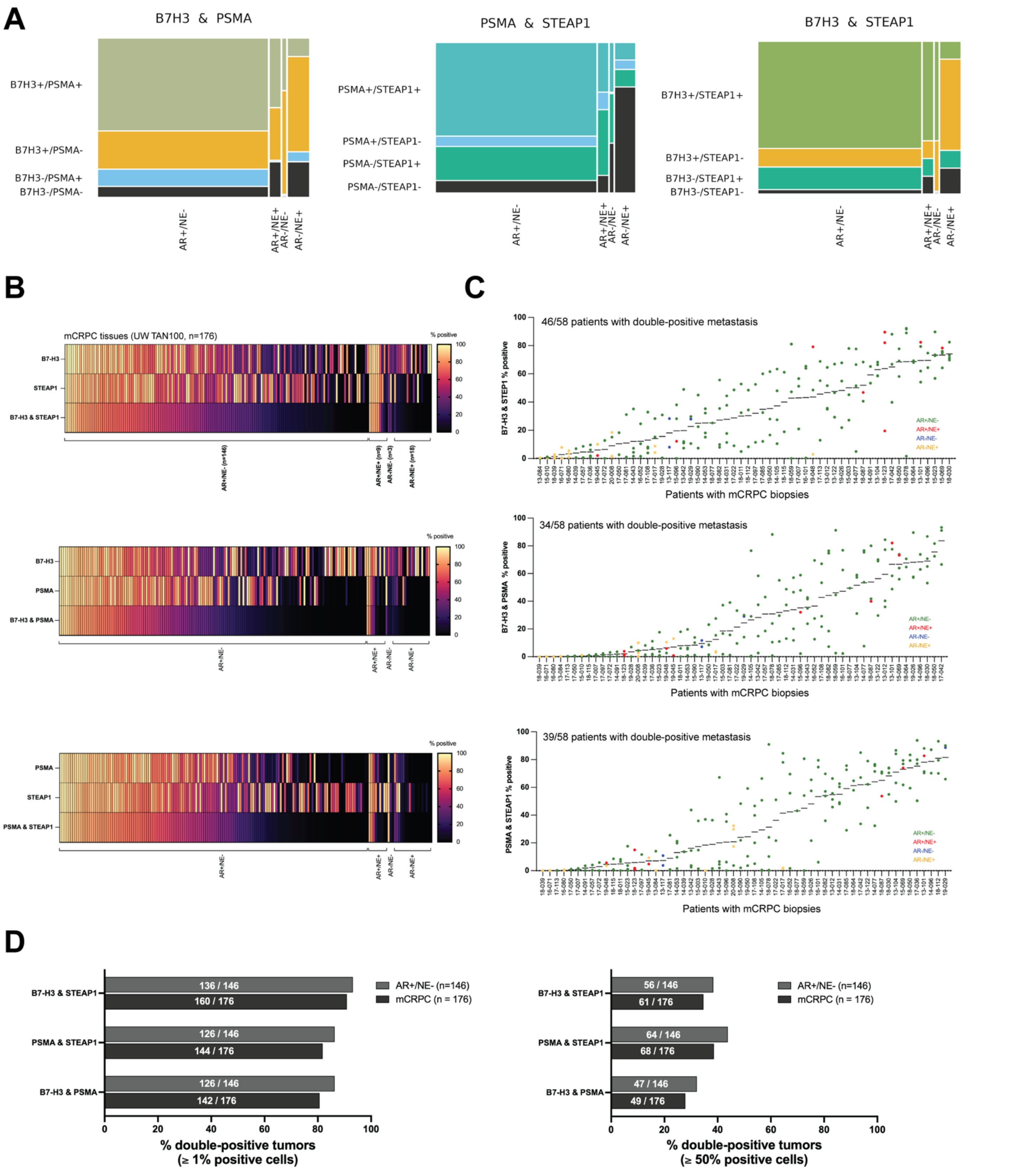

Figure S2. Co-expression of B7-H3 and PSMA / PSMA and STEAP1 / B7-H3 and STEAP1 in mCRPC tumors and patients.

(A) Mosaic plot showing mCRPC subtypes scaled to their relative proportions versus marker status pairs scaled to their relative proportions within each subtype. Double positivity is defined as ≥20% positive cells. (B) Heatmaps showing percents of cells staining positively for B7-H3 and STEAP1, B7-

H3 and PSMA, or PSMA and STEAP1 in each individual mCRPC tumor (columns, n=176). (C) Distribution of B7-H3 and STEAP1, B7-H3 and PSMA, or PSMA and STEAP1 double-positive cells in 176 metastatic tumors within and between 58 patients from UW TAN cohort. Each dot represents a tumor sample; the color codes indicate the molecular subtype – AR+/NE- (green), AR+/NE+ (red), AR-/NE- (blue), and AR-/NE+ (yellow). (D) The percentage of mCRPC or AR+/NE- tumors co-staining for each antigen pair shown for 1% (left) and 50% (right) cutoffs.

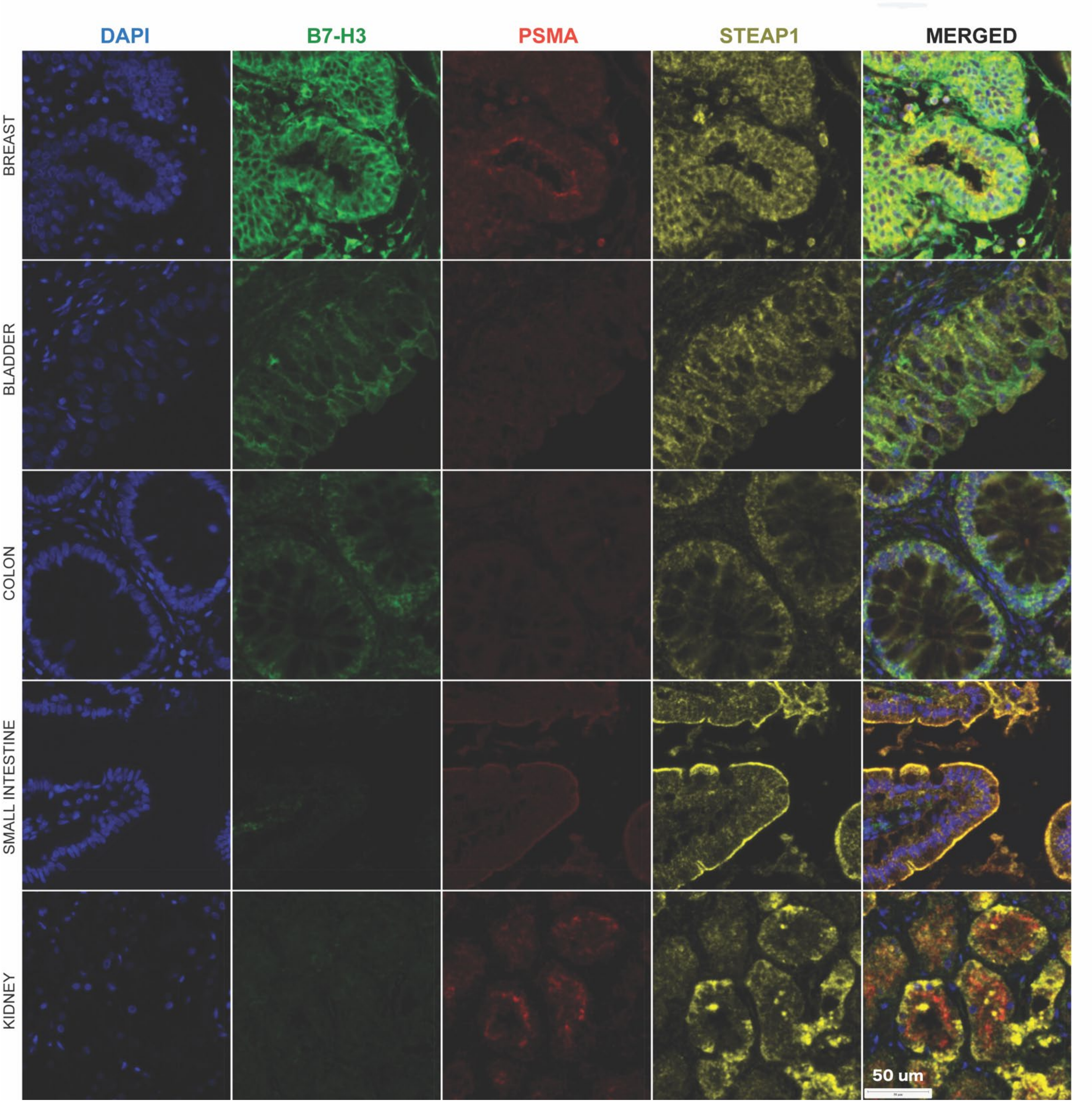

Figure S3. Normal tissues co-expressing B7-H3, PSMA, and STEAP1.

Representative TMA images of human breast, bladder, kidney, small and large intestine tissues (FDA999 L206) with membranous B7-H3, PSMA, STEAP1, and nuclear DAPI staining.

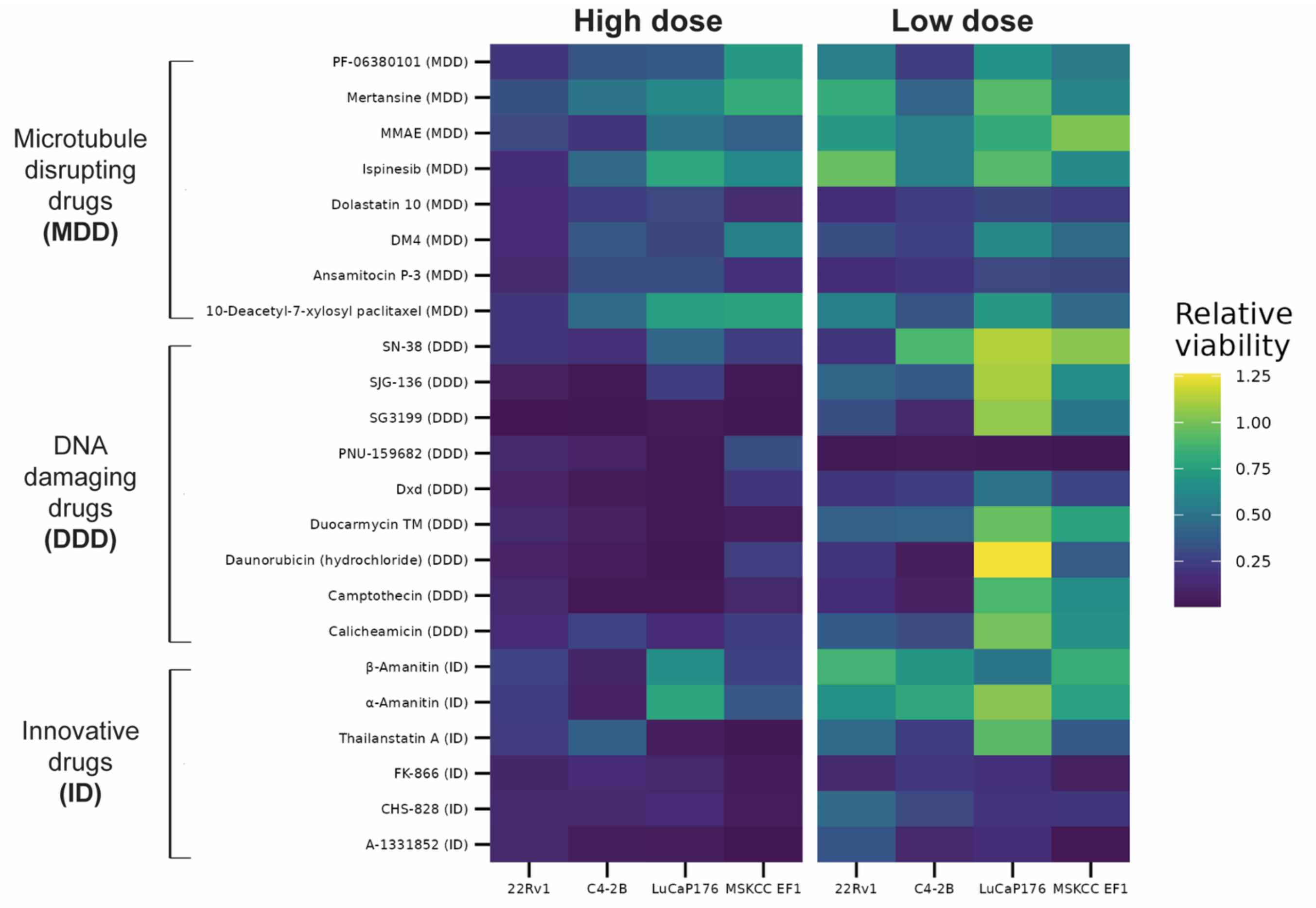

Line	Group 1	Group 2	n 1	n 2	High dose		Low dose	
					**Estimate (95% CI)**	**P-value**	**Estimate (95% CI)**	**P-value**
**C4-2B**	MDD	DDD	8	9	0.09 (0.03, 0.17)	0.005	0.13 (-0.10, 0.28)	>0.9
	MDD	ID	8	6	0.03 (-0.08, 0.10)	0.5	0.01 (-0.44, 0.26)	>0.9
	DDD	ID	9	6	-0.07 (-0.14, 0.00)	0.18	-0.10 (-0.46, 0.15)	>0.9
**22Rv1**	MDD	DDD	8	9	0.28 (0.17, 0.41)	<0.001	0.31 (-0.05, 0.55)	0.2
	MDD	ID	8	6	0.23 (0.09, 0.36)	0.02	0.07 (-0.31, 0.43)	0.8
	DDD	ID	9	6	-0.06 (-0.13, 0.03)	0.11	-0.24 (-0.47, 0.03)	0.2
**LuCaP176**	MDD	DDD	8	9	0.36 (0.26, 0.60)	0.003	-0.27 (-0.58, 0.02)	0.18
	MDD	ID	8	6	0.24 (-0.27, 0.57)	0.18	0.13 (-0.24, 0.63)	0.5
	DDD	ID	9	6	-0.10 (-0.64, 0.03)	0.18	0.41 (-0.05, 0.88)	0.2
**MSKCC EF1**	MDD	DDD	8	9	0.41 (0.13, 0.59)	0.02	-0.06 (-0.33, 0.26)	>0.9
	MDD	ID	8	6	0.44 (0.14, 0.72)	0.02	0.22 (-0.30, 0.52)	>0.9
	DDD	ID	9	6	0.02 (-0.12, 0.21)	0.6	0.23 (-0.19, 0.60)	>0.9

Figure S4. Prostate cancer cell lines demonstrate greater response to DNA-damaging drugs (DDD) compared to microtubule-disrupting drugs (MDD).

Heatmap (top) visualizes relative viability by drug and cell line for high and low doses. RLU – relative luminescence units (relative cell viability). Pairwise comparisons of relative cell viability between payload groups (bottom). The groups were compared using Wilcoxon-Mann-Whitney test.

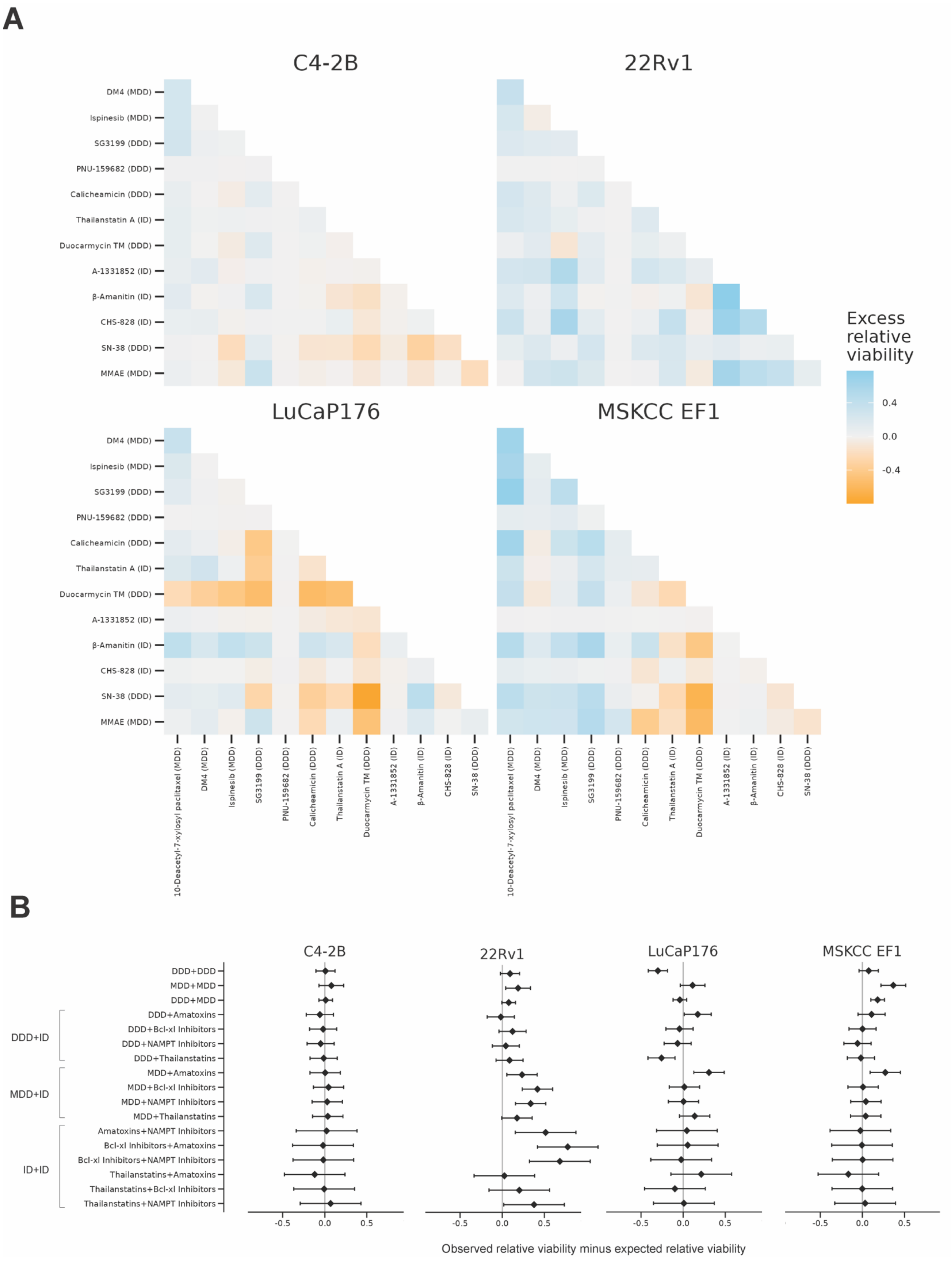
A
C4-2B
22Rv1
LuCaP176
MSKCC EF1
DM4 (MDD)
Ispinesib (MDD)
SG3199 (DDD)
PNU-159682 (DDD)
Calicheamicin (DDD)
Thailanstatin A (ID)
Duocarmycin TM (DDD)
A-1331852 (ID)
β-Amanitin (ID)
CHS-828 (ID)
SN-38 (DDD)
MMAE (MDD)
10-Deacetyl-7-xylosyl paclitaxel (MDD)
Excess relative viability
0.4
0.0
-0.4
B
C4-2B
22Rv1
LuCaP176
MSKCC EF1
DDD+DDD
MDD+MDD
DDD+MDD
DDD+Amatoxins
DDD+Bcl-xl Inhibitors
DDD+NAMPT Inhibitors
DDD+Thailanstatins
MDD+Amatoxins
MDD+Bcl-xl Inhibitors
MDD+NAMPT Inhibitors
MDD+Thailanstatins
Amatoxins+NAMPT Inhibitors
Bcl-xl Inhibitors+Amatoxins
Bcl-xl Inhibitors+NAMPT Inhibitors
Thailanstatins+Amatoxins
Thailanstatins+Bcl-xl Inhibitors
Thailanstatins+NAMPT Inhibitors
DDD+ID
MDD+ID
ID+ID
-0.5
0.0
0.5
Observed relative viability minus expected relative viability

Figure S5. Combination payload screening prioritizes candidates for synergy assessment.

(A) Heatmaps showing excess relative viability of C4-2B, 22Rv1, LuCaP 176, and MSKCC EF1 cells exposed to single payloads at low dose and payload combinations. Excess viability was calculated as *observed viability* ($viability_{drug1+drug2}$) - *expected viability* ($viability_{drug1}$ * $viability_{drug2}$). (B). Excess relative viability means for the combinations between payload groups and classes in four cell lines.

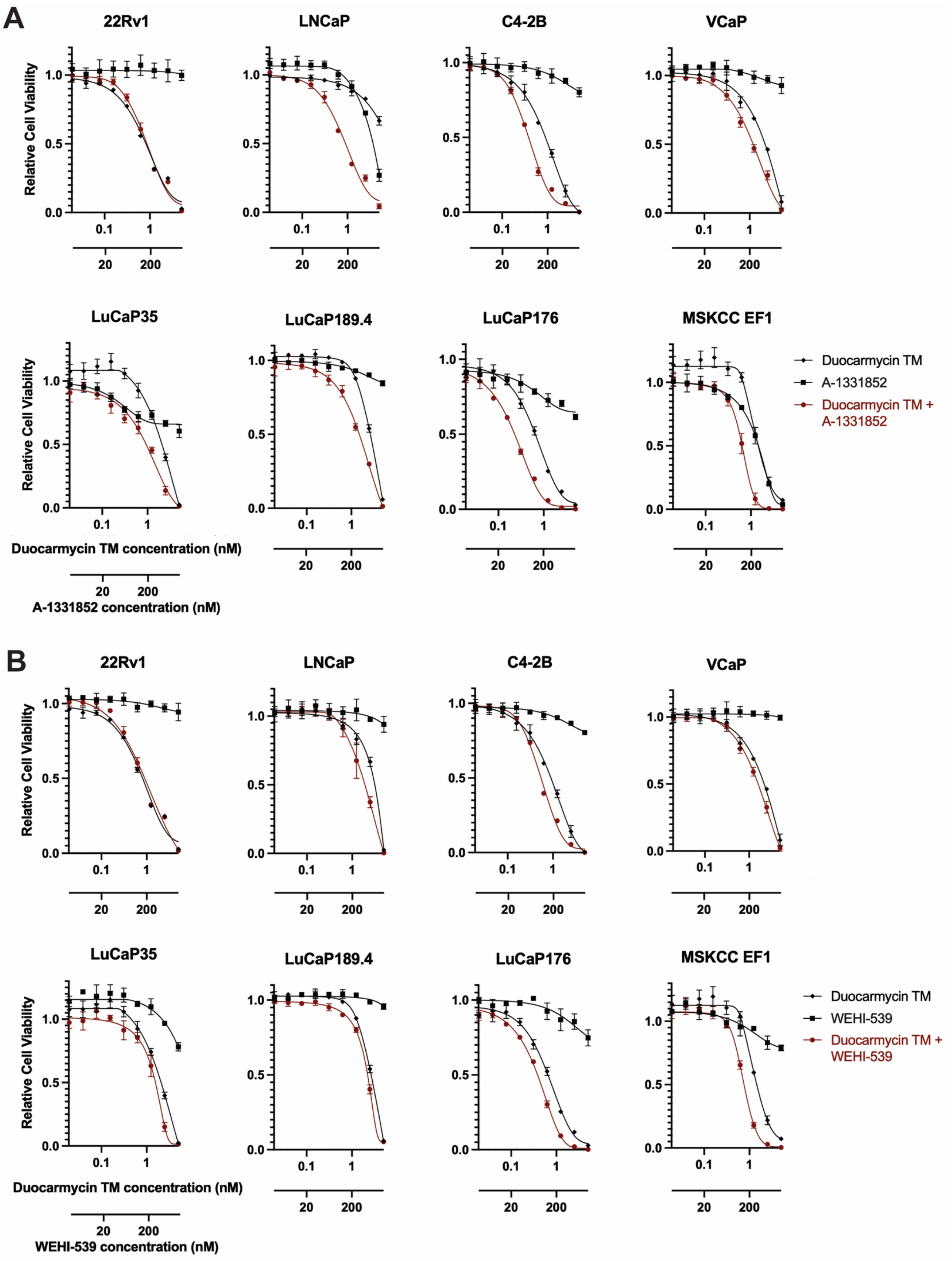

A
22Rv1
LNCaP
C4-2B
VCaP
LuCaP35
LuCaP189.4
LuCaP176
MSKCC EF1
Relative Cell Viability
Duocarmycin TM
A-1331852
Duocarmycin TM + A-1331852
Duocarmycin TM concentration (nM)
A-1331852 concentration (nM)
B
22Rv1
LNCaP
C4-2B
VCaP
LuCaP35
LuCaP189.4
LuCaP176
MSKCC EF1
Relative Cell Viability
Duocarmycin TM
WEHI-539
Duocarmycin TM + WEHI-539
Duocarmycin TM concentration (nM)
WEHI-539 concentration (nM)

Figure S6. Duocarmycin TM and BCL-XL inhibitors (A-1331852 and WEHI-539) combinations exhibit synergistic cytotoxicity in a panel of prostate cancer cell lines.

(A) Relative viability of PC cells exposed to Duocarmycin TM and A-1331852. (B) Relative viability of PC cells exposed to Duocarmycin TM and WEHI-539.

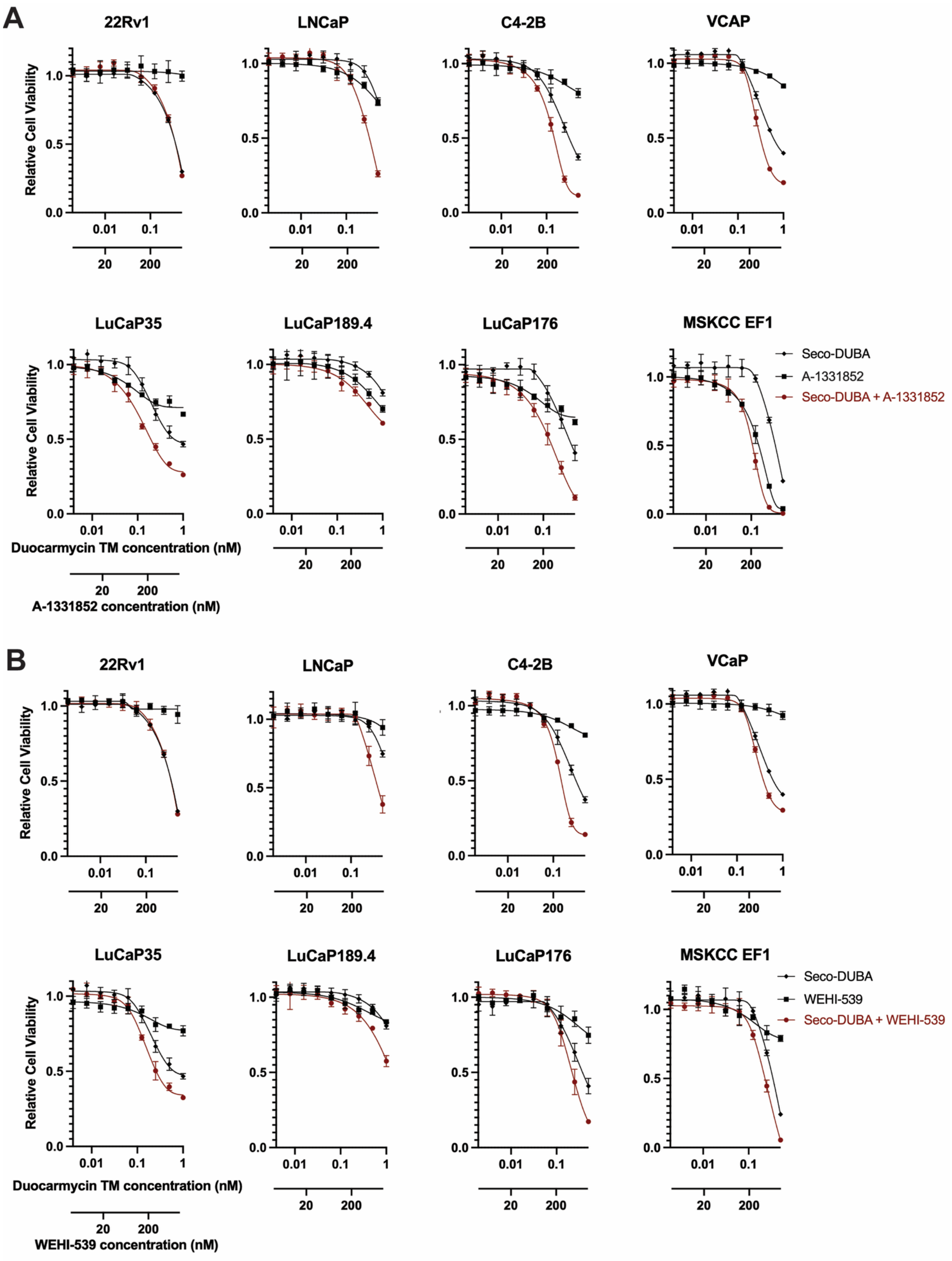
A
22Rv1
LNCaP
C4-2B
VCAP
LuCaP35
LuCaP189.4
LuCaP176
MSKCC EF1
Relative Cell Viability
Duocarmycin TM concentration (nM)
A-1331852 concentration (nM)
Seco-DUBA
A-1331852
Seco-DUBA + A-1331852
B
22Rv1
LNCaP
C4-2B
VCaP
LuCaP35
LuCaP189.4
LuCaP176
MSKCC EF1
Relative Cell Viability
Duocarmycin TM concentration (nM)
WEHI-539 concentration (nM)
Seco-DUBA
WEHI-539
Seco-DUBA + WEHI-539

Figure S7. Seco-DUBA and BCL-XL inhibitors (A-1331852 and WEHI-539) combinations exhibit synergistic cytotoxicity in a panel of prostate cancer cell lines.

(A) Relative viability of PC cells exposed to seco-DUBA and A-1331852. (B) Relative viability of PC cells exposed to seco-DUBA and WEHI-539.

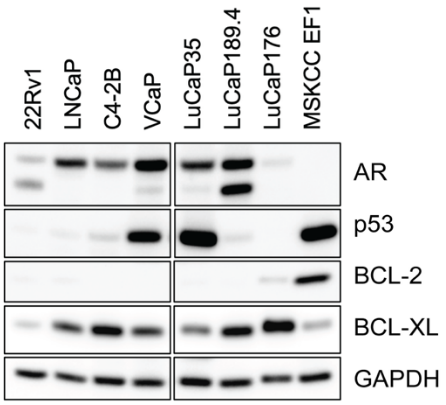

Figure S8. Immunoblot analysis of AR, p53, BCL-2 and BCL-XL protein levels in 8 PC cell lines.

A

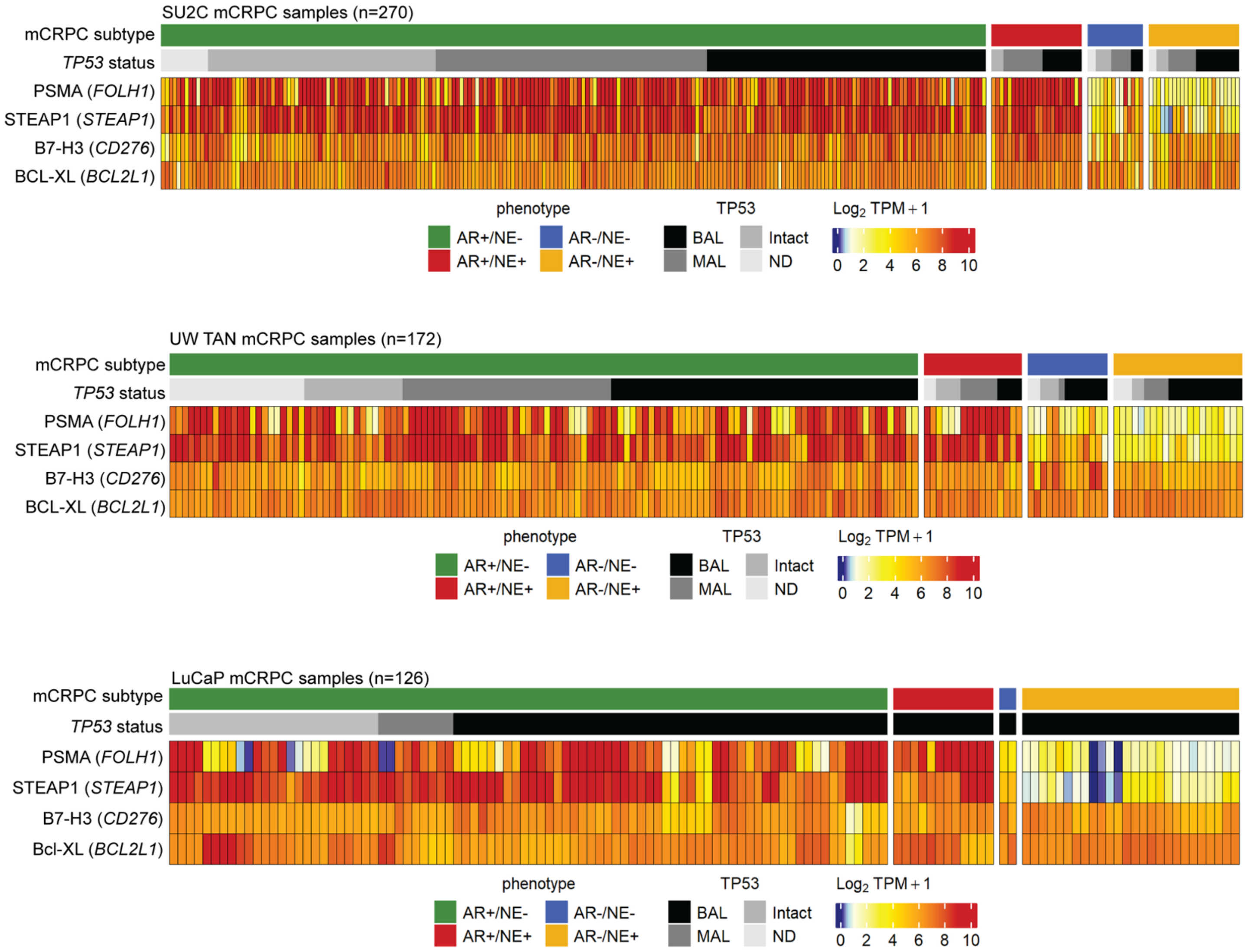

B

Dataset	BAL	Intact	MAL	ND	Total	%BAL
SU2C	95	68	91	16	270	35%
AR+/NE-	71	58	69	12	210	34%
AR+/NE+	10	3	10	0	23	43%
AR-/NE-	3	4	5	2	14	21%
AR-/NE+	11	3	7	2	23	48%
UW TAN	73	25	45	29	172	42%
AR+/NE-	50	16	34	22	122	41%
AR+/NE+	4	4	6	2	16	25%
AR-/NE-	7	3	1	2	13	54%
AR-/NE+	12	2	4	3	21	57%
LuCaP	92	25	9	0	126	73%
AR+/NE-	52	25	9	0	86	60%
AR+/NE+	12	0	0	0	12	100%
AR-/NE-	2	0	0	0	2	100%
AR-/NE+	26	0	0	0	26	100%

Figure S9. *TP53* genomic alterations in mCRPCs expressing B7-H3 (*CD276*), PSMA (*FOLH1*), STEAP1, and BCL-XL (*BCL2L1*).

(A) Heatmap showing *FOLH1, STEAP1, CD276,* and *BCL2L1* transcript abundance, as well as *TP53* genomic status in SU2C, UW TAN and LuCaP mCRPC specimens. Transcript levels are shown as Log_2 TPM + 1. BAL – biallelic loss, MAL – monoallelic loss, ND – no data. (B) Fractions of tumors with and without TP53 genomic alterations in SU2C, UW TAN, and LuCaP cohorts across 4 mCRPC molecular subtypes.

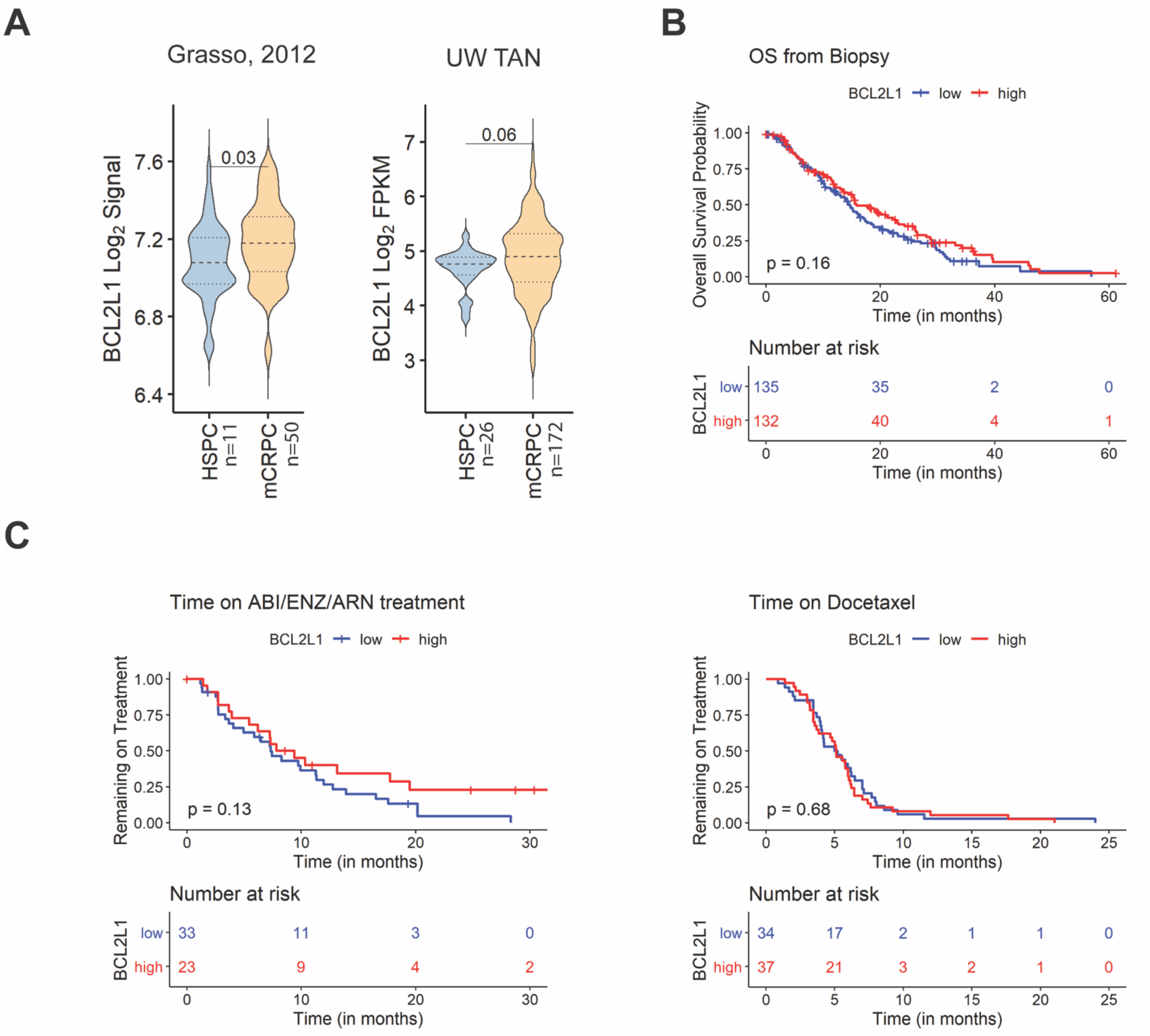

Figure S10. BCL-XL/*BCL2L1* expression effects on disease stage and SoC therapy response.

(A) Violin plots showing BCL-XL/*BCL2L1* transcript levels in HSPC and mCRPC from Grasso 2012 and UW cohorts. The groups were compared using two-sided Wilcoxon rank tests. (B) Kaplan-Meier estimates of patient overall survival (OS) from the time of biopsy in SU2C mCRPC dataset. (C) Kaplan-Meier estimates of time on treatment for AR signaling inhibitors (Abiraterone, Enzalutamide, or Apalutamide; left) and Docetaxel (right) in SU2C mCRPC dataset Patients were stratified into "high" and "low" groups based on median *BCL2L1* expression. Survival distributions were compared using the log-rank test.

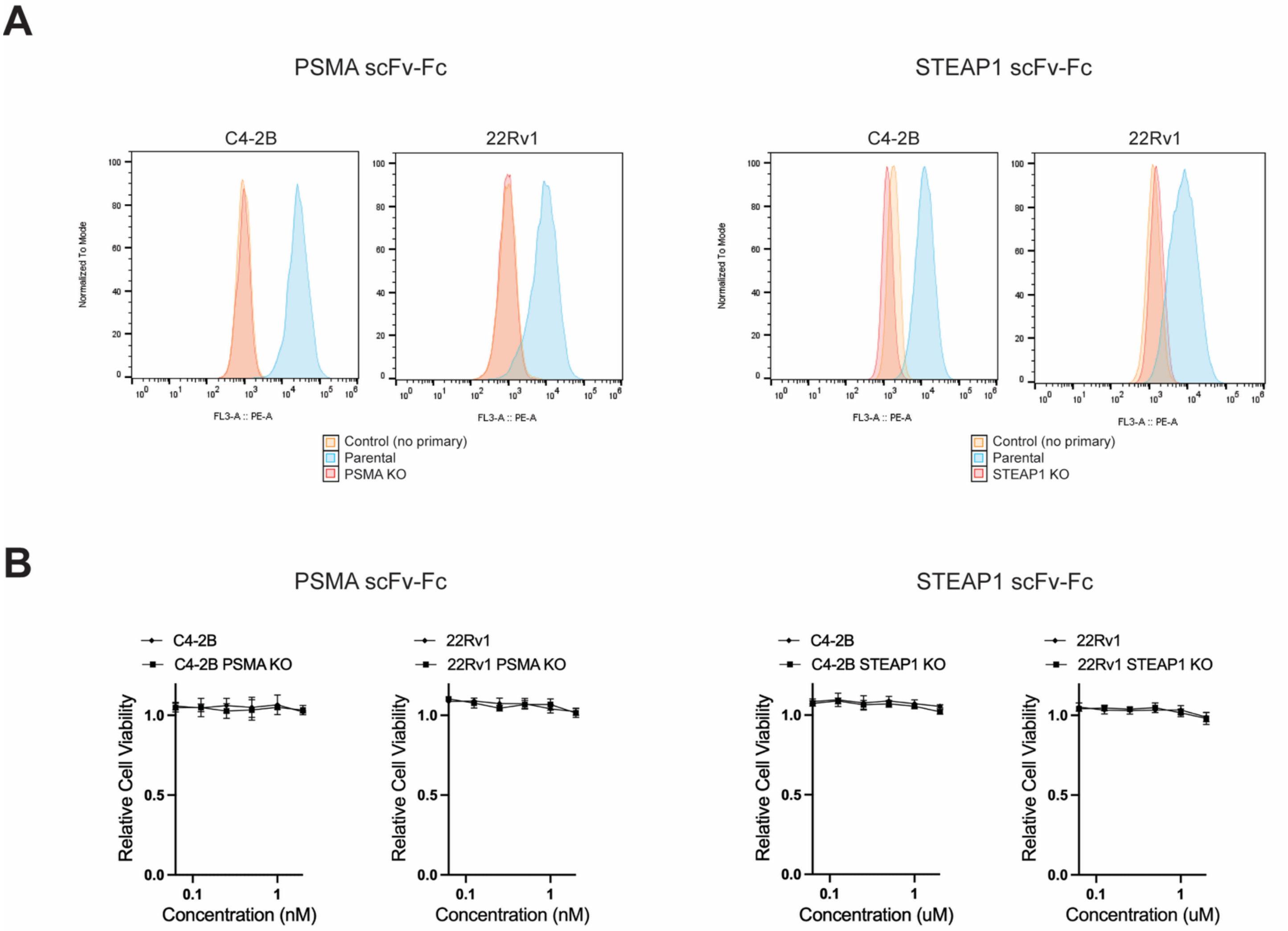

Figure S11. Quality control of PSMA and STEAP1 scFv-Fc antibodies.

(A) PSMA scFv-Fc or STEAP1 scFv-Fc binding to the target antigens determined by flow cytometry in parental and antigen KO C4-2B and 22Rv1 cells. (B) Viability of C4-2B and 22Rv1 cells exposed to naked PSMA scFv-Fc or STEAP1 scFv-Fc.

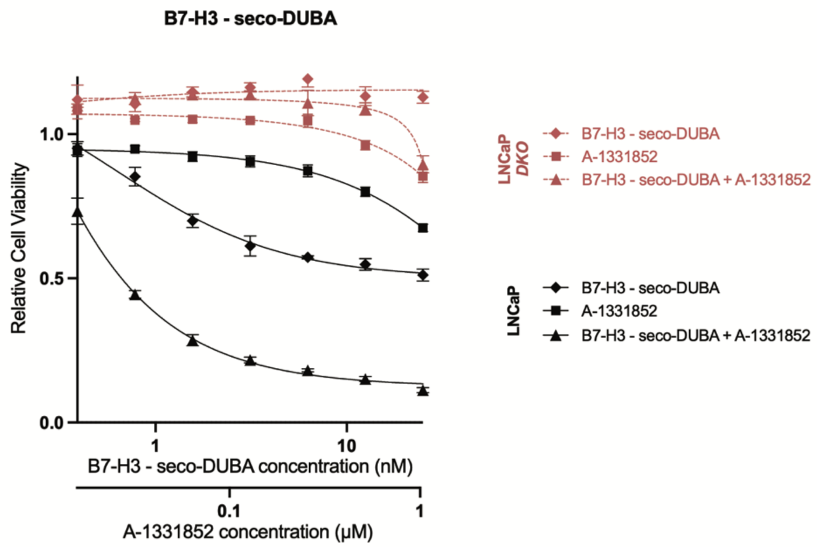

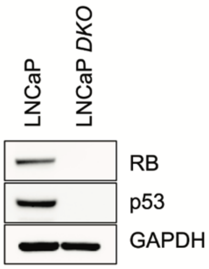

Figure S12. LNCaP *RB1 KO/TP53 KO* (LNCaP DKO) cells demonstrate resistance to B7-H3 – seco-DUBA (MGC018), A-1331852, and the combination *in vitro*.

Dose-response to MGC018, A-1331852, and the combination in LNCaP and LNCaP *DKO* cells (left) and immunoblot showing *RB1* and *TP53* knockout in LNCaP *DKO* (right).

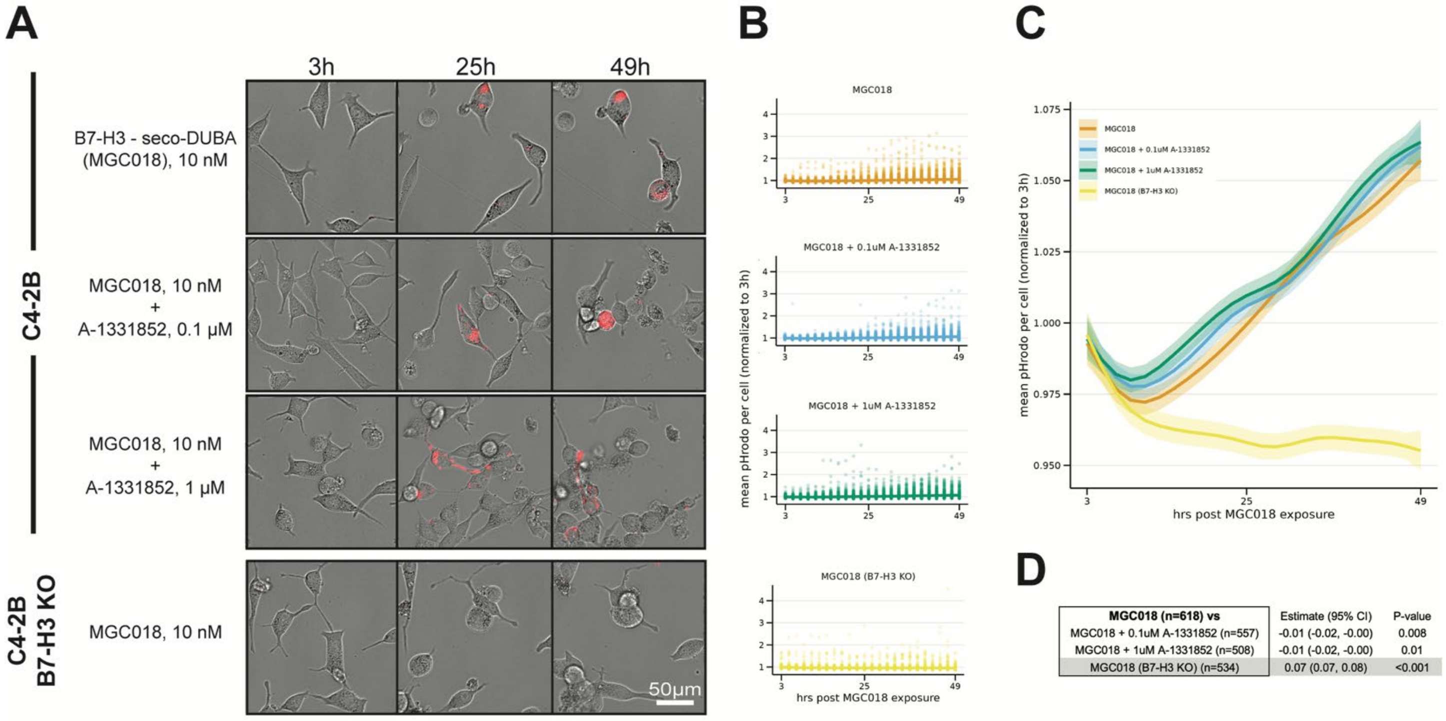

**MGC018 (n=618) vs**	Estimate (95% CI)	P-value
MGC018 + 0.1uM A-1331852 (n=557)	-0.01 (-0.02, -0.00)	0.008
MGC018 + 1uM A-1331852 (n=508)	-0.01 (-0.02, -0.00)	0.01
MGC018 (B7-H3 KO) (n=534)	0.07 (0.07, 0.08)	<0.001

Figure S13. Effects of A-1331852 on membrane trafficking of B7-H3 – seco-DUBA (MGC018).

(A) Representative images demonstrating internalization of MGC018 into C4-2B (in the absence or presence of A-1331852) and C4-2B B7-H3 KO cells. (B-D) Estimated temporal trends in normalized mean pHrodo intensity following MGC018 exposure from a generalized additive model with a factor–smooth interaction between time and treatment group using a Gamma distribution with log link and fit using restricted maximum likelihood. B) Group-stratified fits with raw observations (points) superimposed to illustrate the underlying data distribution. C) Estimated mean trajectories by group with Wald-type pointwise 95% confidence intervals (shaded bands) computed from the model-based standard errors on the link scale and back-transformed to the response scale. Confidence bands reflect uncertainty in the estimated mean trajectories but do not adjust for multiple comparisons across time points. D) Median differences at the 49-hour endpoint between MGC018-treated C4-2B cells and other experimental groups, with significance determined by Wilcoxon-Mann-Whitney tests.

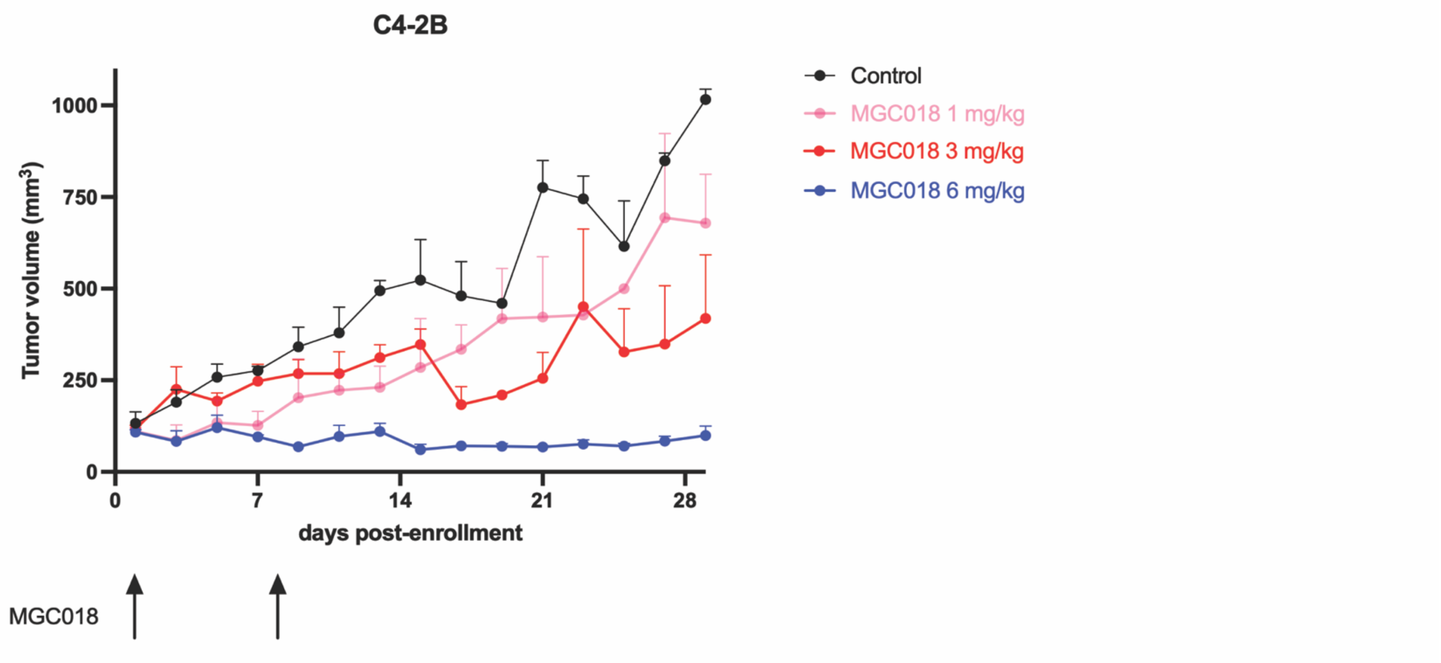

Figure S14. Volumetric changes in C4-2B CDX tumors (n=3) in animals treated with vehicle control or MGC018 at 1-6 mg/kg (IP, weekly).

A

Group * Day	exp(Beta)	95% CI[1]	p-value	exp(Beta)	95% CI[1]	p-value
B7-H3 – seco-DUBA* Day	0.95	0.94, 0.96	<0.001	0.97	0.96, 0.98	<0.001
A1331852 * Day	0.97	0.96, 0.98	<0.001	0.99	0.98, 1.00	0.065
B7-H3 – seco-DUBA+A1331852 * Day	0.94	0.93, 0.94	<0.001	0.96	0.95, 0.96	<0.001
	C4-2B			C4-2B *TP53 KO*		

B

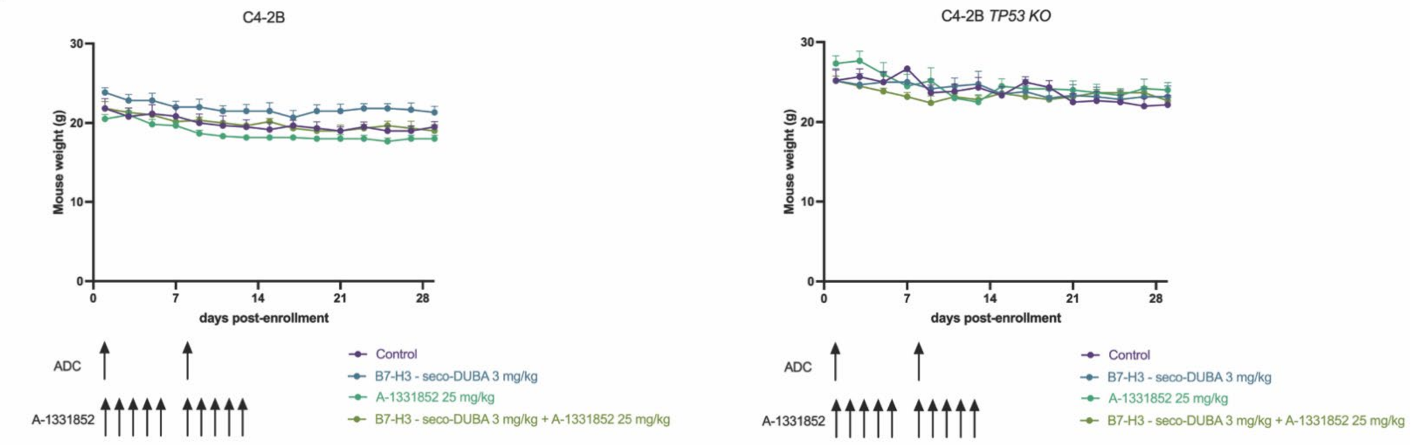

C

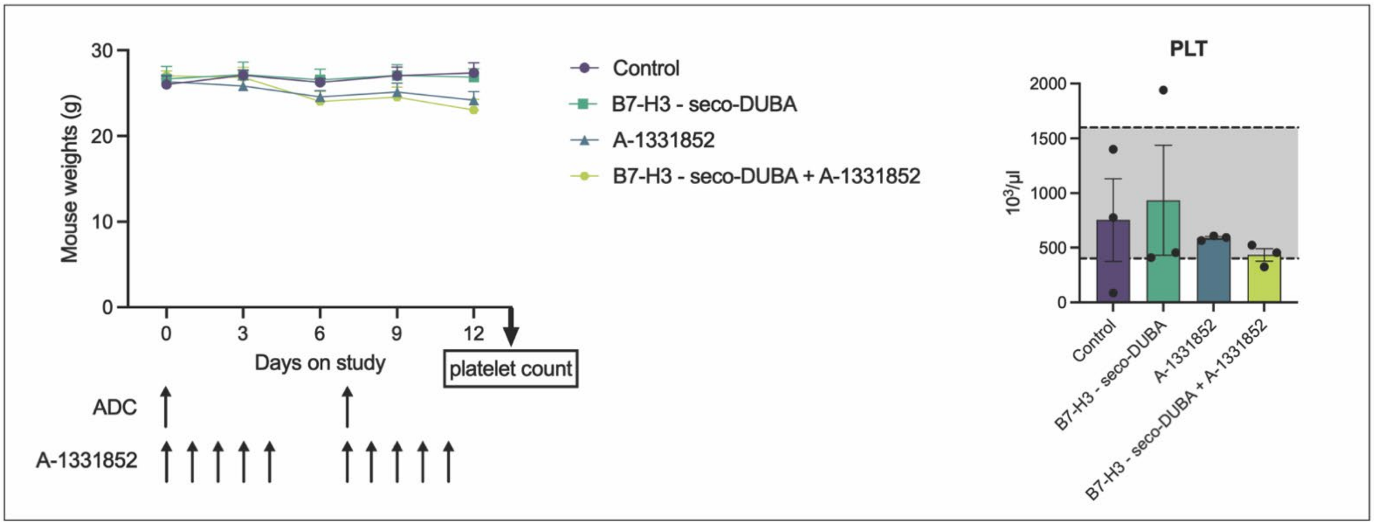

Figure S15. Analysis of volumetric changes in CDX tumors and body weight changes in animals from control and treatment C4-2B and C4-2B *TP53 KO* groups.

(A) Statistical analysis for the estimated tumor volume growth rate with tests relative to the control. CI[1] – confidence interval. (B) Average body weights per group throughout the experiment. (C) Average body weights (left) and platelet counts (right) in the toxicity experiment. Gray shading indicates the standard physiological range. Error bars represent standard error of the mean.

Table S1. Cytotoxic agents used in the drug screen.

Compound name	Vendor	Catalog #	References	Concentration (high) in uM	Concentration (low) in uM
10-Deacetyl-7-xylosyl paclitaxel	MedChemExpress	HY-20584	*Jiang et al., 2008*	5	0.5
PF-06380101	MedChemExpress	HY-12522	*Akaiwa et al., 2018*	0.001	0.0001
Mertansine	MedChemExpress	HY-19792	*Widdison et al., 2006*	0.001	0.0001
DM4	MedChemExpress	HY-12454	*Widdison et al., 2006*	0.005	0.0005
Ispinesib	MedChemExpress	HY-50759	*Purcell et al., 2010*	0.005	0.0005
SG3199	MedChemExpress	HY-101161	*Tiberghien et al., 2016*	0.001	0.0001
SJG-136	MedChemExpress	HY-14573	*Hartley et al., 2004*	0.005	0.0005
PNU-159682	MedChemExpress	HY-16700	*Quintieri et al., 2005*	0.001	0.0001
Daunorubicin (hydrochloride)	MedChemExpress	HY-13062	*Kalashnikova et al., 2025*	1	0.1
DXd	MedChemExpress	HY-13631D	*Ogitani et al.,*	1	0.1
Calicheamicin	MedChemExpress	HY-19609	*Boghaert et al., 2004*	0.001	0.0001
Thailanstatin A	MedChemExpress	HY-129589	*Liu et al., 2013*	1	0.1
Duocarmycin TM	MedChemExpress	HY-107769	*Boger et al., 1994*	0.001	0.0001
Ansamitocin P-3	Sigma-Aldrich	A2836	*Venghateri et al. 2013*	5	0.5
Camptothecin	Sigma-Aldrich	C9911	*Tesauro et al., 2019*	1	0.1
Dolastatin 10	Sigma-Aldrich	CS-1825	*Bai et al., 1990*	1	0.1
A-1331852	Cayman Chemical	22963	*Rahman et al., 2022*	5	0.5
α-Amanitin	Cayman Chemical	17898	*Moldenhauer et al., 2012*	0.5	0.05
β-Amanitin	Cayman Chemical	18142	*Bang et al., 2022*	0.5	0.05
FK-866	Cayman Chemical	13287	*Hasmann et al., 2003*	0.5	0.05
CHS-828	Cayman Chemical	11021	*Olesen et al., 2008*	0.05	0.005
SN-38	Selleckchem	S4908	*Jonsson et al., 2000*	0.1	0.01
MMAE	Selleckchem	S7721	*Doronina et al., 2003*	0.001	0.0001

Table S2. List of primary and secondary antibodies used for multiplexed immunofluorescent staining.

Protein target	Antibody	Host/clone	Manufacturer/ Cat#	Concentration/ Dilution	Secondary/ Cat#	Opal Dye/ Cat#
1	**STEAP1**	Rabbit polyclonal	LS Bio LS-C291740	2µg/ml 1:500	1X Opal Anti-Ms + Rb HRP ARH1001EA	570 FP1488001KT
2	**PSMA**	Mouse 3E6	Agilent M3620	0.157µg/ml 1:1000	1X Opal Anti-Ms + Rb HRP ARH1001EA	690 FP1497001KT
3	**B7-H3**	Rabbit EPR20115	Abcam ab219648	0.038 µg/ml 1:15,000	1X Opal Anti-Ms + Rb HRP ARH1001EA	520 FP1487001KT

Table S5. Dose-response to BCL-XL inhibitor A-1331852, DDDs SN-38, PNU-159682, Calicheamicin, SG-3199, Duocarmycin TM, and 5 combinations of DDDs with A-1331852 in LNCaP cells.

CI – combination index.

CI	22Rv1	LNCaP	C4-2B	VCaP	LuCaP35	LuCaP189.4	LuCaP176	MSKCC EF1
Duocarmycin TM + A1331852	1.15	0.12	0.62	0.45	0.30	0.26	0.34	0.52
Duocarmycin TM + WEHI-539	1.50	0.54	0.76	0.60	0.59	0.52	0.48	0.34
Seco-DUBA + A1331852	1.10	0.63	0.50	0.77	0.53	0.72	0.14	0.58
Seco-DUBA + WEHI-539	1.12	0.57	0.63	0.8	0.58	0.56	0.42	0.44

Table S6. Components of genotoxic ADCs targeting B7-H3, PSMA, and STEAP1.

ADC Name	ADC Target	Antibody Format	ADC Payload	ADC Linker	DAR
B7-H3 – seco-DUBA (MGC018) (vobramitamab duocarmazine) (vobra duo)	**B7-H3**	IgG1	Seco-DUBA	Val-Cit	2.7
PSMA – SG3249	**PSMA**	scFv-Fc	SG3249 (Tesirine)	Mal-PEG8-Val-Ala-PABC	3
STEAP1 - DXd	**STEAP1**	scFv-Fc	DXd	MC-GGFG	1.09

Table S7. Protein sequences of PSMA and STEAP1 targeted scFv-Fcs.

PSMA (J591) scFv-Fc	METDTLLLWVLLLLAAQPAMAEVQLVQSGPEVKKPGATVKISCKTSGYTFTEYTIHWV<br>KQAPGKGLEWIGNINPNNGGTTYNQKFEDKATLTVDKSTDTAYMELSSLRSEDTAVYY<br>CAAGWNFDYWGQGTLLTVSSGSTSGGGSGGGSGGGGSSDIQMTQSPSSLSTSVGD<br>RVTLTCKASQDVGTAVDWYQQKPGQSPKLLIYWASTRHTGIPSRFSGSGSGTDFTLTI<br>SSLQPEDFADYYCQQYNSYPLTFGAGTKVDIKGPGGPEPKSSDKTHTCPPCPAPELL<br>GGPSVFLFPPKPKDTLMISRTPEVTCVVVDVSHEDPEVKFNWYVDGVEVHNAKTKPR<br>EEQYNSTYRVVSVLTVLHQDWLNGKEYKCKVSNKALPAPIEKTISKAKGQPREPQVYT<br>LPPSRDELTKNQVSLTCLVKGFYPSDIAVEWESNGQPENNYKTTPPVLDSDGSFFLYS<br>KLTVDKSRWQQGNVFSCSVMHEALHNHYTQKSLSLSPGK*
STEAP1 (DSTP3086S)<br>scFv-Fc	METDTLLLWVLLLLAAQPAMADIQMTQSPSSLSASVGDRVTITCKSSQSLLYRSNQKN<br>YLAWYQQKPGKAPKLLIYWASTRESGVPSRFSGSGSGTDFTLTISSLQPEDFATYYCQ<br>QYYNYPRTFGQGTKVEIKRGGGGSGGGGSGGGGSEVQLVESGGGLVQPGGSLRLS<br>CAVSGYSITSDYAWNWVRQAPGKGLEWVGYISNSGSTSYNPSLKSRFTISRDTSKNTL<br>YLQMNSLRAEDTAVYYCARERNYDYDDYYYAMDYWGQGTLVTVSSGPGGPEPKSSD<br>KTHTCPPCPAPELLGGPSVFLFPPKPKDTLMISRTPEVTCVVVDVSHEDPEVKFNWYV<br>DGVEVHNAKTKPREEQYNSTYRVVSVLTVLHQDWLNGKEYKCKVSNKALPAPIEKTIS<br>KAKGQPREPQVYTLPPSRDELTKNQVSLTCLVKGFYPSDIAVEWESNGQPENNYKTT<br>PPVLDSDGSFFLYSKLTVDKSRWQQGNVFSCSVMHEALHNHYTQKSLSLSPGK*
