# Supplementary material for: Genotoxic antibody-drug conjugates combined with BCL-XL inhibitors enhance therapeutic efficacy in metastatic castration-resistant prostate cancer
Source: J Clin Invest. 2026 Jun 23;136(15):e200438. doi: 10.1172/JCI200438 (PMC13430021; doi:10.1172/JCI200438)

Full unedited blots for Figure 3F.

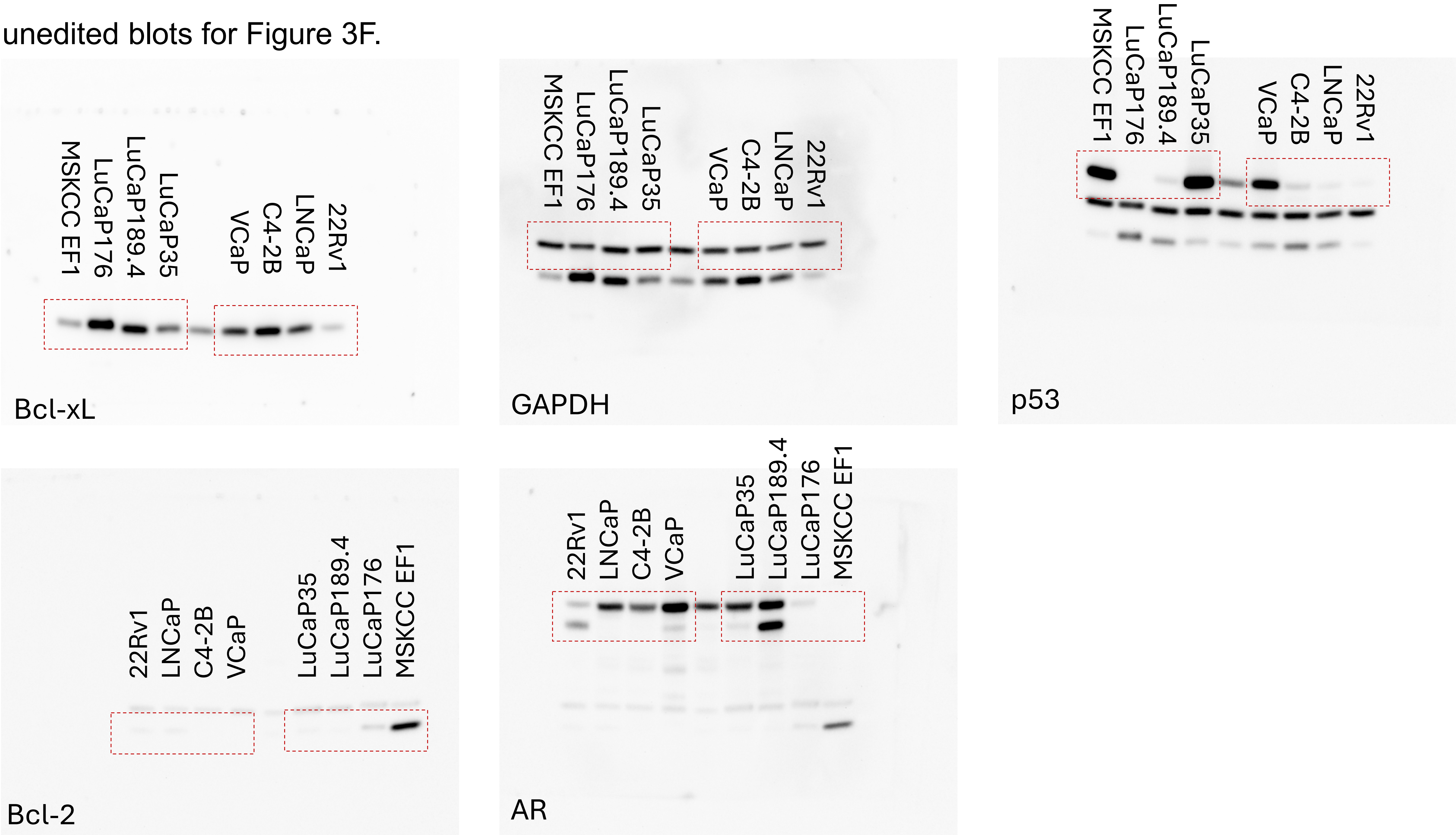

Full unedited blots for Figure 4D (LNCaP).

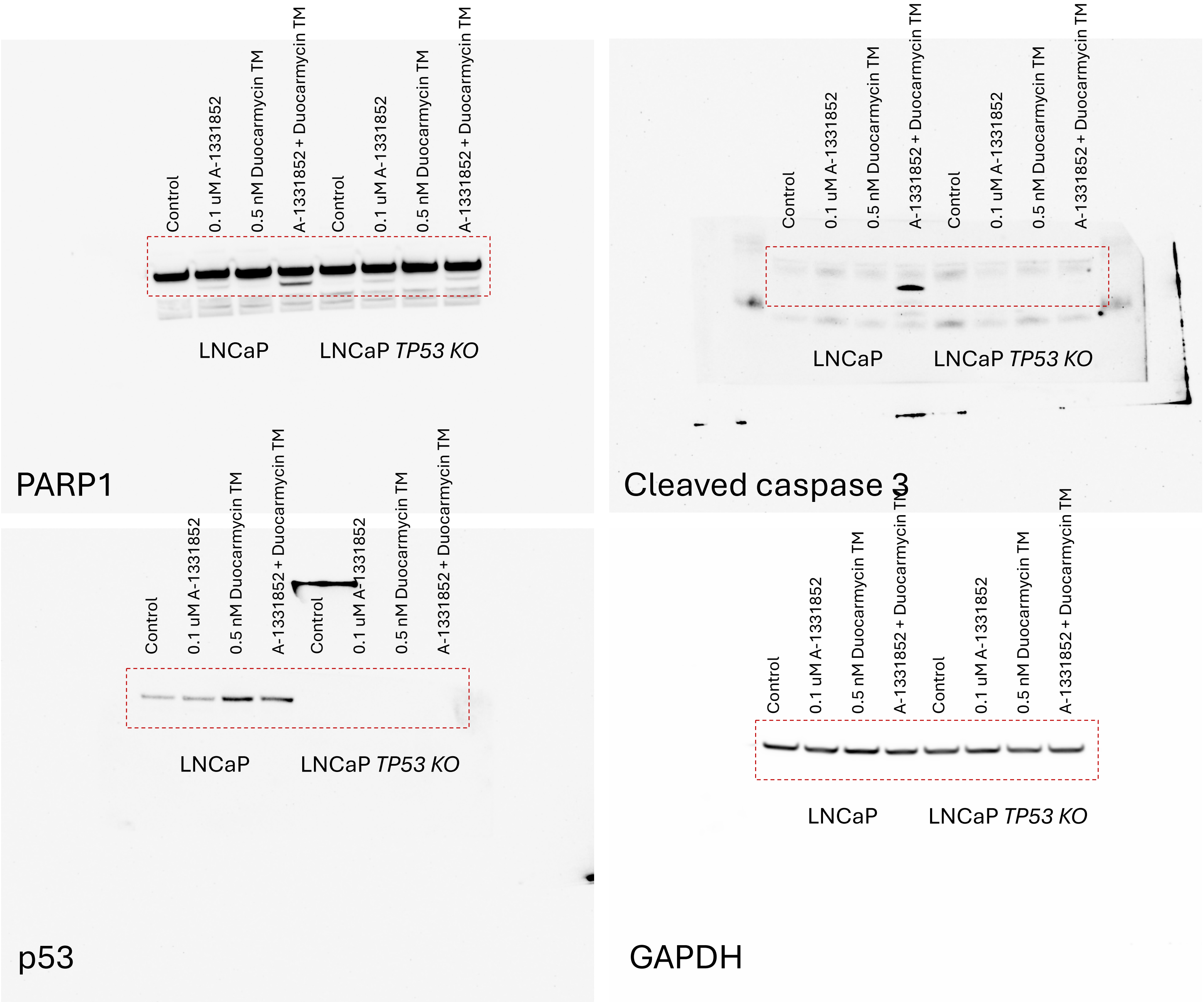

Full unedited blot for Figure 4D (C4-2B).

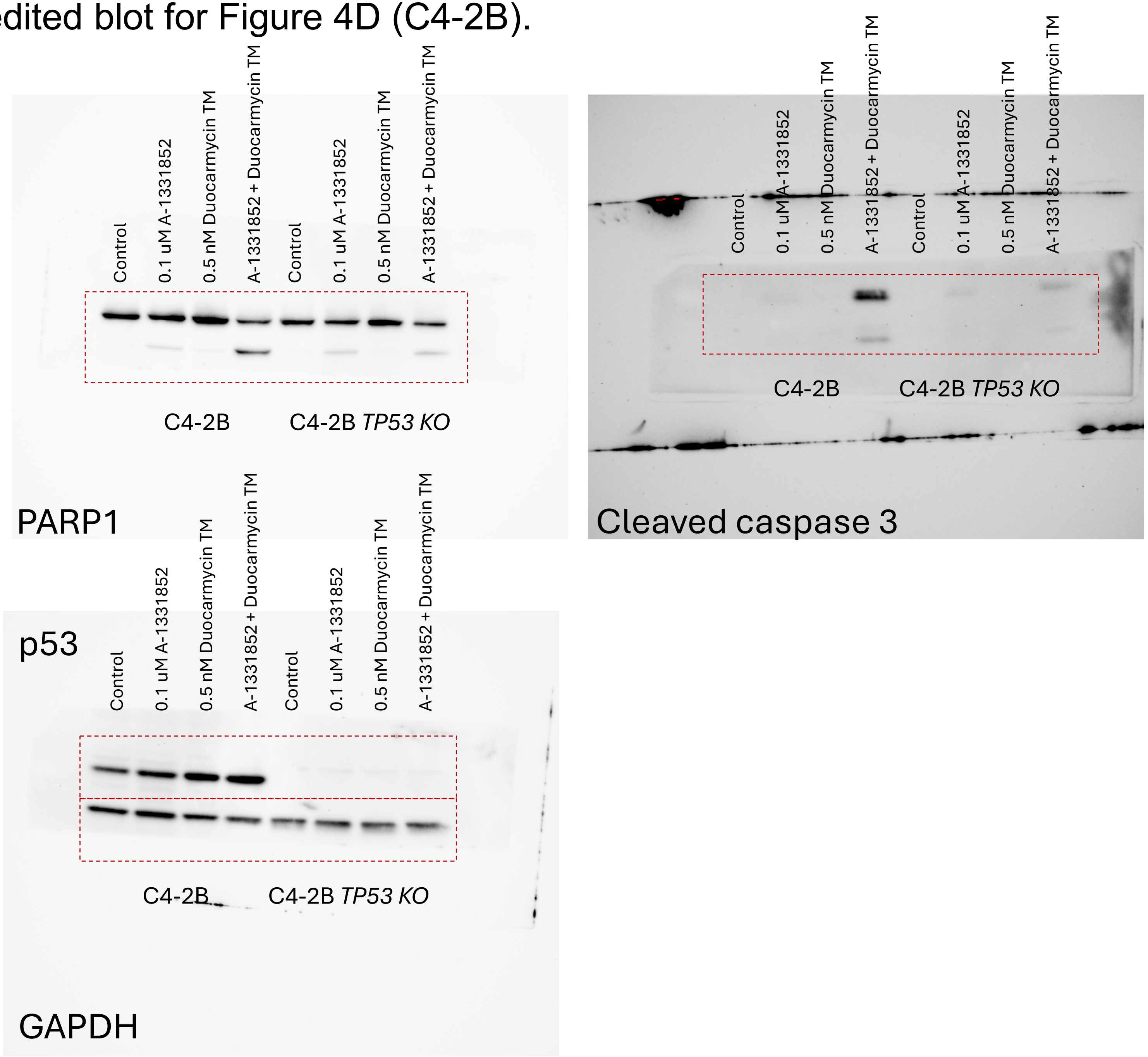

Full unedited blots for Figure 4D (LuCaP189.4).

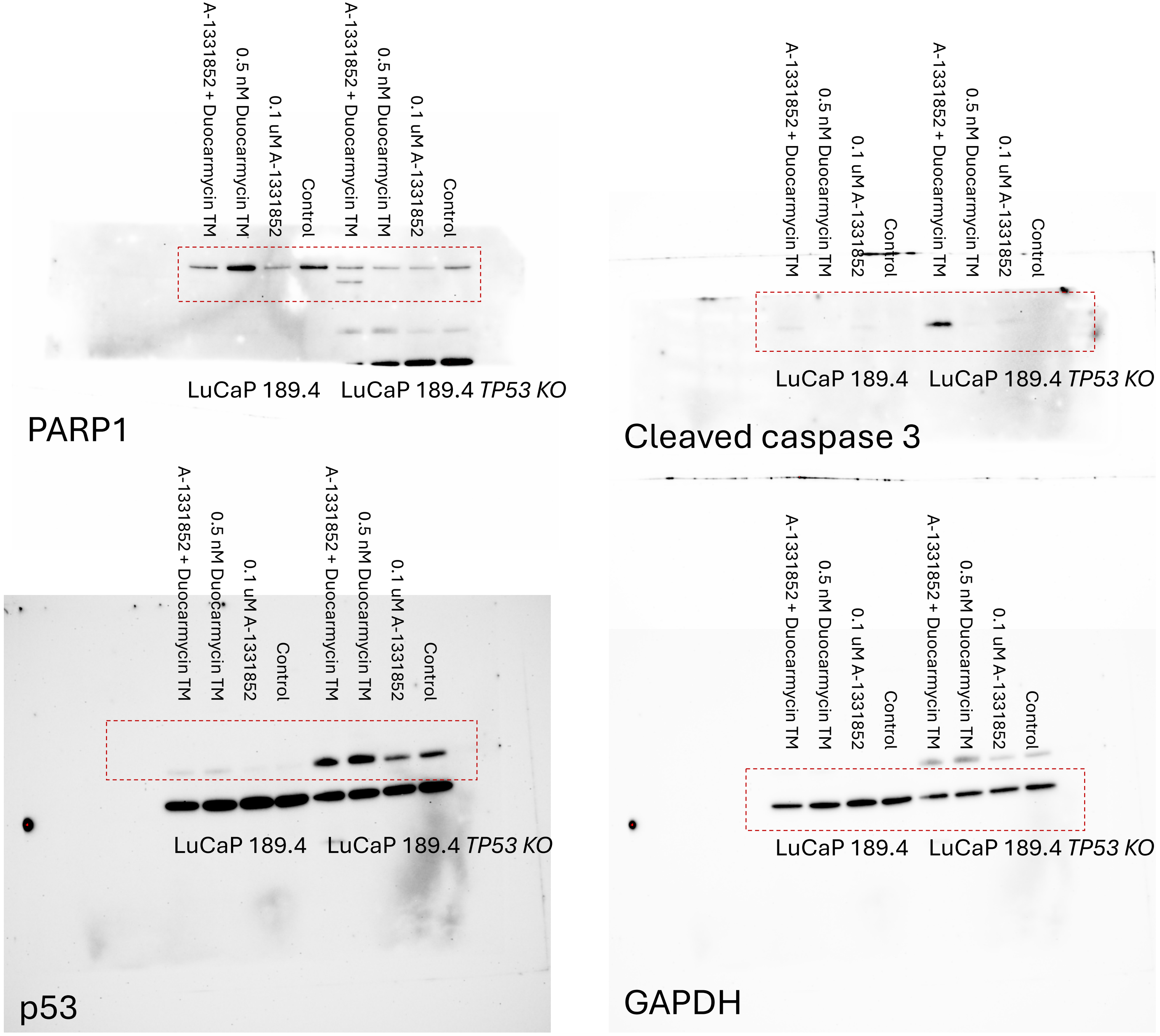

Full unedited blots for Figure 5A.

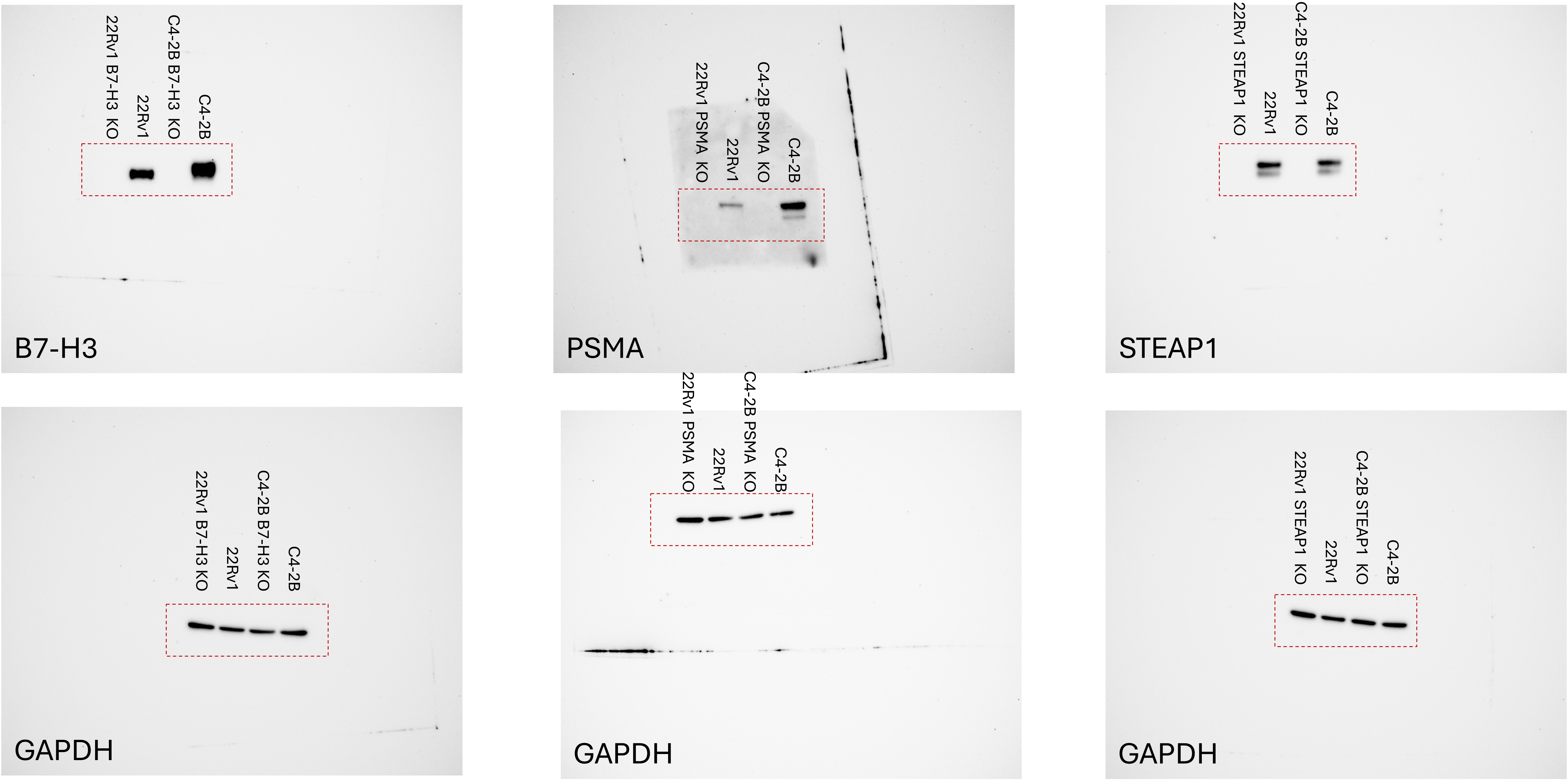

Full unedited blots for Figure 5B (B7-H3 – seco-DUBA).

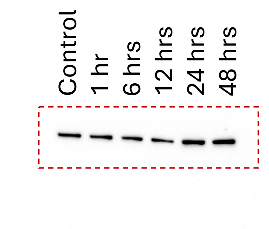

PARP1

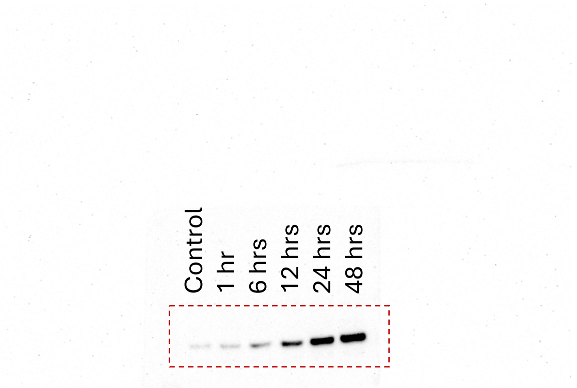

p53

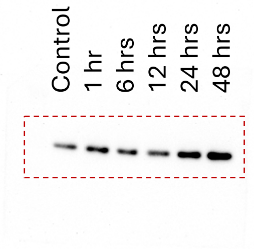

Bcl-xL

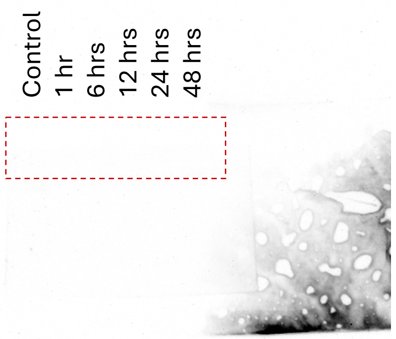

Bcl-2

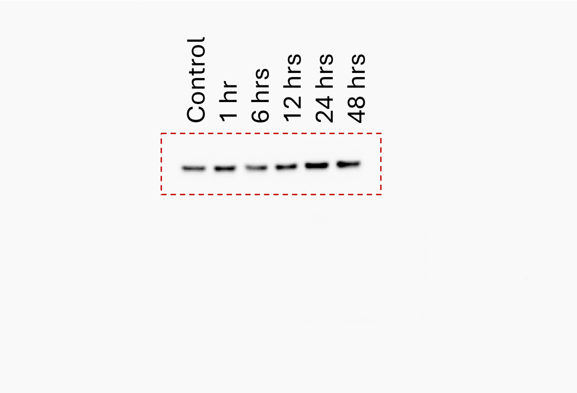

Mcl-1

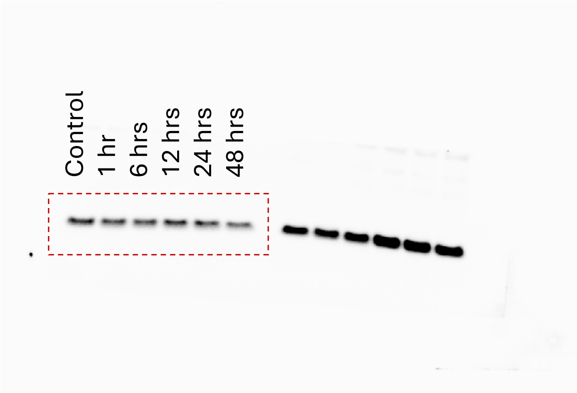

Bcl-w

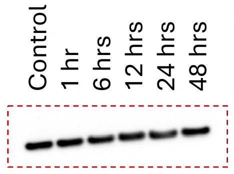

GAPDH

Full unedited blots for Figure 5B (PSMA– SG3249).

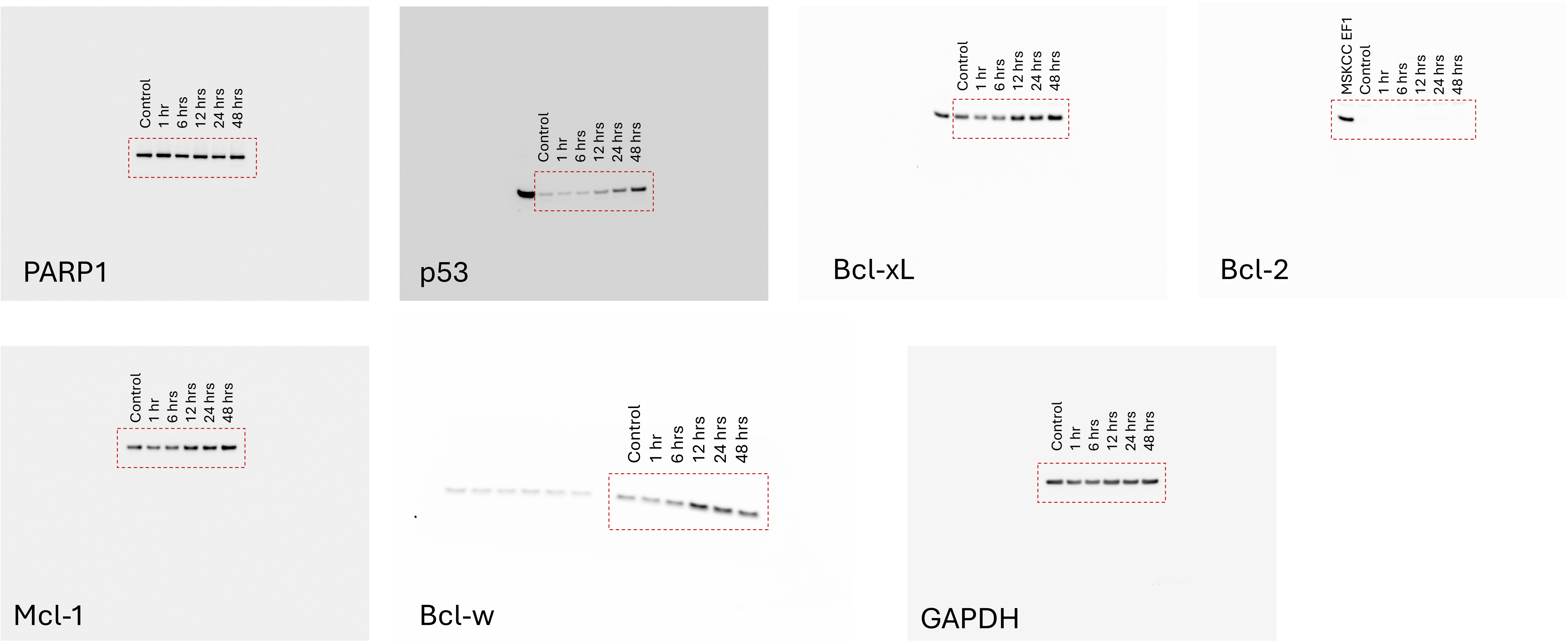

Full unedited blots for Figure 5B (STEAP1– DXd).

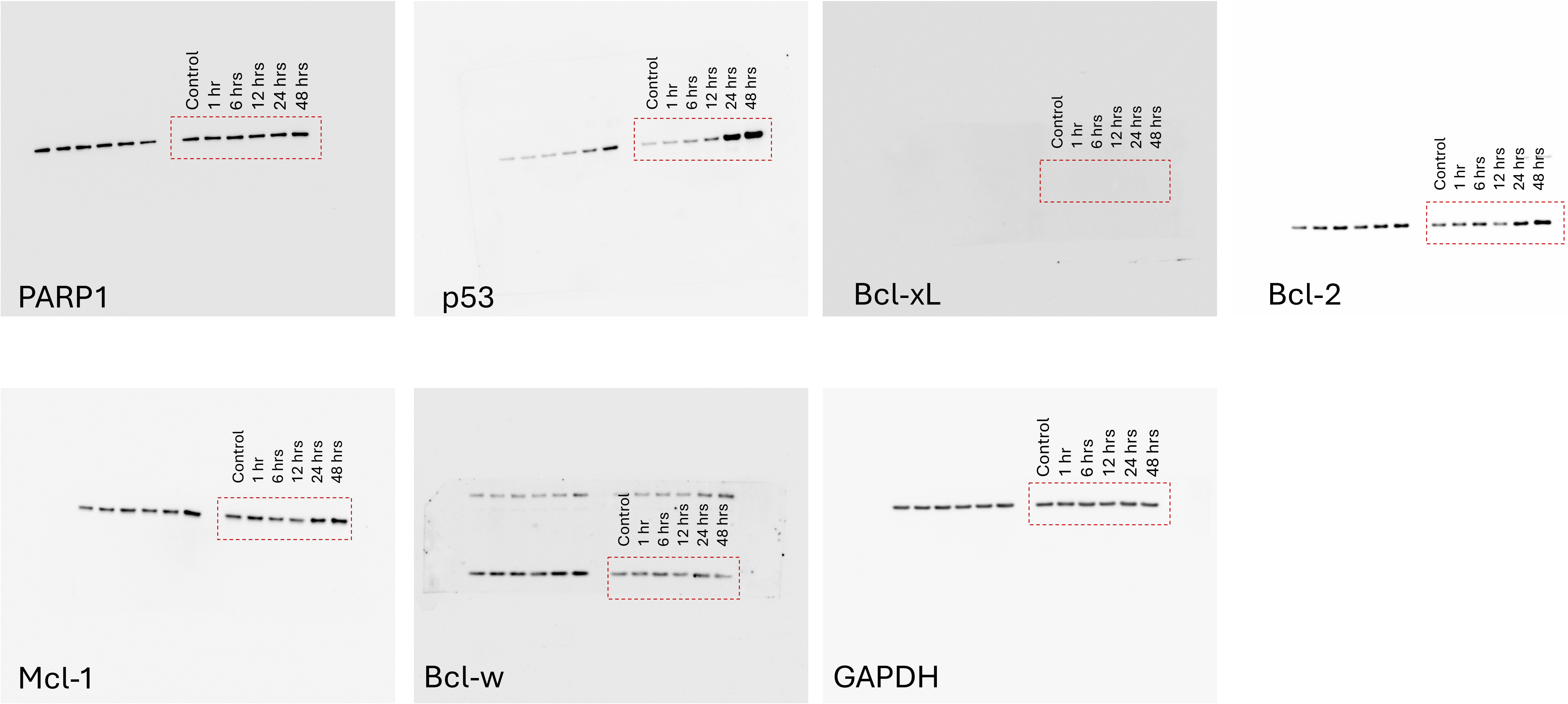

Full unedited blots for Figure S11.

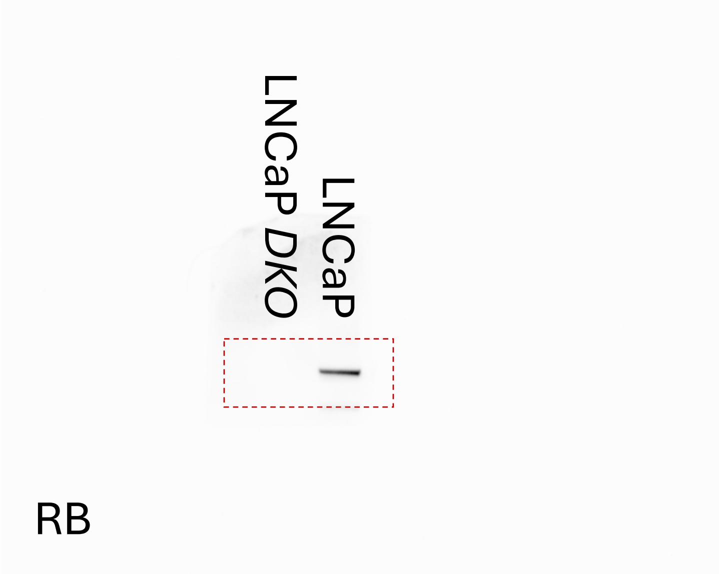

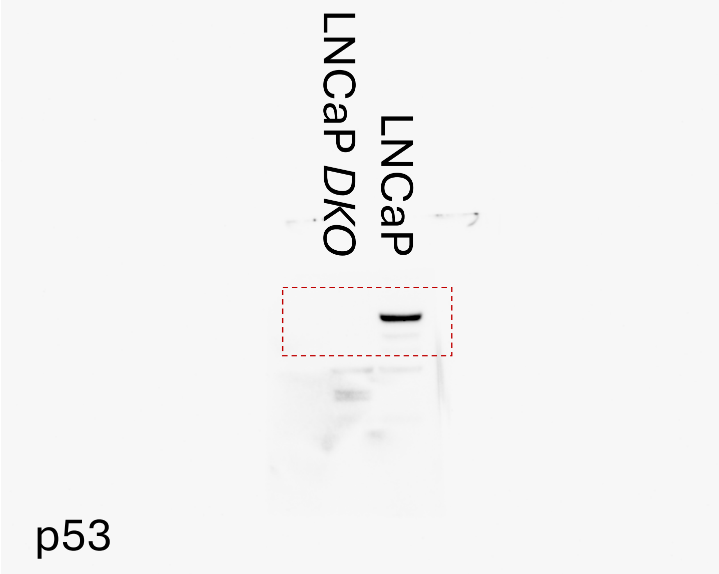

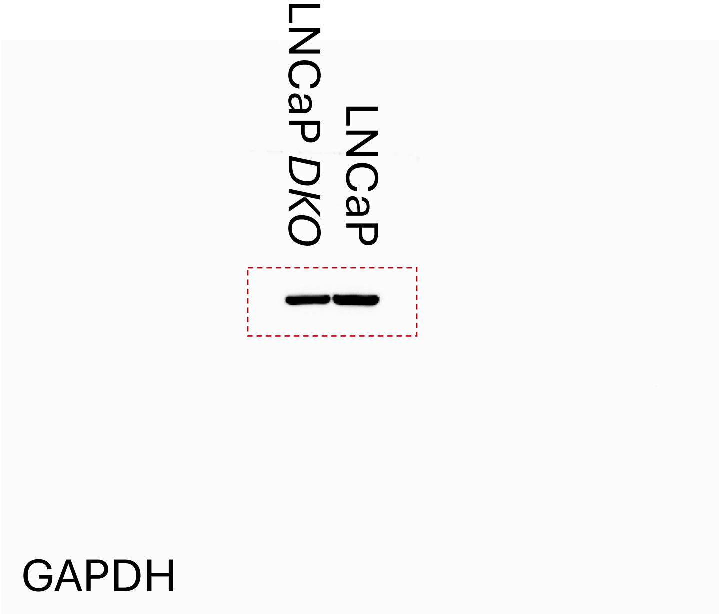

Supplement: Unedited blot and gel images [file jci-136-200438-s069.pdf]
